# Alternative splicing regulates the expression of G9A and SUV39H2 methyltransferases, and dramatically changes SUV39H2 functions

**DOI:** 10.1093/nar/gkv013

**Published:** 2015-01-20

**Authors:** Oriane Mauger, Roscoe Klinck, Benoit Chabot, Christian Muchardt, Eric Allemand, Eric Batsché

**Affiliations:** 1 Sorbonne Universités, Université Pierre et Marie Curie, Université Paris 6, IFD, 4 Place Jussieu, 75252 PARIS cedex 05, France; 2 Institut Pasteur, Département de Biologie du Développement et Cellules Souches, CNRS URA2578, Unité de Régulation Epigénétique, 25 rue du Docteur Roux, Paris, 75015, France; 3 Laboratory of Functional Genomics of the Université de Sherbrooke, Sherbrooke, Québec, J1E 4K8, Canada; 4 Department of Microbiology and Infectious Diseases, Faculty of Medicine and Health Sciences, Université de Sherbrooke, Sherbrooke. Québec, J1E 4K8, Canada

## Abstract

Alternative splicing is the main source of proteome diversity. Here, we have investigated how alternative splicing affects the function of two human histone methyltransferases (HMTase): G9A and SUV39H2. We show that exon 10 in *G9A* and exon 3 in *SUV39H2* are alternatively included in a variety of tissues and cell lines, as well as in a different species. The production of these variants is likely tightly regulated because both constitutive and alternative splicing factors control their splicing profiles. Based on this evidence, we have assessed the link between the inclusion of these exons and the activity of both enzymes. We document that these HMTase genes yield several protein isoforms, which are likely issued from alternative splicing regulation. We demonstrate that inclusion of *SUV39H2* exon 3 is a determinant of the stability, the sub-nuclear localization, and the HMTase activity. Genome-wide expression analysis further revealed that alternative inclusion of *SUV39H2* exon 3 differentially modulates the expression of target genes. Our data also suggest that a variant of *G9A* may display a function that is independent of H3K9 methylation. Our work emphasizes that expression and function of genes are not collinear; therefore alternative splicing must be taken into account in any functional study.

## INTRODUCTION

Alternative splicing affects the sequence of mature RNAs and is a source of proteome diversity. From transcriptome analysis, it appears that the impact of splicing on gene expression has been underestimated and recent studies suggest that almost every gene gives rise to alternatively spliced transcripts (1,2). Most of these alternative splicing events have not been characterized, and the study of their function represents a major challenge for biology.

Pre-mRNA splicing is a stepwise process catalyzed by the spliceosome, a macromolecular machinery composed of five snRNPs and approximately a hundred splicing factors (3,4). This enzymatic complex is highly dynamic, allowing flexible pathways to regulate splicing. Intron removal by the spliceosome does not strictly require other RNA processing machineries (5–7). Nevertheless, it is now accepted that regulation of splicing is influenced by both transcription and chromatin (8–10). In particular, the transcription machinery is important for the loading of early splicing factors on nascent transcripts (11,12), while chromatin may directly or indirectly affect recruitment of splicing factors to sites of transcription (13–18). Conversely, splicing factors can also influence transcription (19,20) and splicing is now proposed to locally affect chromatin properties (21–25). These observations suggest a crosstalk between splicing and chromatin machineries possibly giving rise to multiple feed back loops involved in regulation of gene expression.

Alternative splicing is also expected to impact the expression and activity of chromatin factors, although it has rarely been considered in functional studies. Here, we have characterized protein isoforms of histone methyltransferases (HMTase), G9A (EHMT2/KMT1C) and SUV39H2. These enzymes belong to a family of six members, which also includes GLP (EHMT1), SETDB1, and SUV39H1. These HMTases cooperate in the control of the mono-, di-, or tri-methylation of histone H3 at lysine 9 (H3K9me1/2/3) (26,27), and these marks are generally associated with transcriptional silencing (28).

G9A is essential for embryonic stem cell differentiation and development, and it catalyzes H3K9me1/2 marks enriched in euchromatin (29,30). Abundant data on G9A activity support its role as a transcriptional repressor. The SUV39H1/H2 enzymes create the H3K9me3 mark, a histone modification mainly localized to pericentromeric regions (31–34). SUV39H2 was originally described as embryonic- and testis-specific (34), but a comparison between Suv39h1/Suv39h2 double null mice and Suv39h1 null mice suggests that SUV39H2 may also have functions in other tissues (35).

In the present study, we demonstrate that exon 10 in *G9A* and exon 3 in *SUV39H2* are alternatively spliced. Inclusion of these exons is controlled in various tissues and cell lines and also regulated in different species. Western blot analysis confirms the existence of several protein isoforms expressed by both *G9A* and *SUV39H2*. Based on this evidence, we have investigated the functional consequences associated with the alternative splicing of these exons. We find that SUV39H2 protein isoforms differ in their stability, chromatin localization, HMTase activities and their impact on gene expression. On the other hand, alternative splicing may confer to G9A an H3K9me-independent function. Our study further extends the interconnections linking splicing, transcription and chromatin, and indicates that alternative splicing plays an important role in expanding the identity and function of histone methyltransferases.

## MATERIALS AND METHODS

### Cell culture and siRNA transfections

HEK 293T, WI38 (ATCC), the ovarian cancer cell lines SW626 (HTB-78, ATCC), SKOV3 cells were obtained from ATCC (HTB-77) and SKOV3-ip previously described (36) were maintained in Dulbecco's modified Eagle's medium (Gibco, #31966–021) supplemented with 10% (v/v) foetal bovine serum (Thermo Scientific, #SV30160.03) and 100 U/ml penicillin-streptomycin (Gibco, #15140–122), HeLa cells (CCL-2, ATCC) were grown in the same medium supplemented with 7% (v/v) foetal bovine serum. Cells were transfected with a mix containing siRNA (10–20 nM) and RNAi Max reagent (Life Technologies, #13778075) according to the reverse transfection protocol then collected 72 h after for analysis. For experiments in which the cells were transfected twice, the second round of transfection was performed 72 h after the first one. G9A and SUV39H2 siRNAs were synthesized as ON-TARGET*plus* grade from Dharmacon, siRNA against RNA-binding protein were synthesized as 5′ phosphorylated RNA by SIGMA. Sequences of siRNA are listed in Supplementary Table S3.

### Tissues and organisms, RNA extraction, reverse transcription, radio-labeled and quantitative real-time PCR

Mouse, Chicken and Zebrafish samples were collected at indicated times and stored at -80C during 4 months. Total RNA from human tissues were from Clontech (#636533 and #636643), and those from cells and organisms were extracted as described (37) followed with DNase I (Roche, #04716728001) treatment. The quality of RNA was checked on agarose gel. The cDNA libraries were built using 0.5 μg RNA reverse transcribed with M-MLV reverse transcriptase (Life Technologies, #28025013) and either oligo dT (Thermo Scientific, #SO131), random hexamers (Sigma) or target specific primers (see Supplementary Table S3). Semi-quantitative polymerase chain reaction (PCR) was performed with γ-^32^P 5′ end-labeled primers, and PCR products were verified by DNA sequencing. Quantitative real-time PCR (qPCR) was assayed in 10 μl reactions with Brillant III Ultra Fast SYBR-Green QPCR Master Mix (Agilent, #600882). PCR reactions were carried out in a Stratagene MX3005p system with the following thermal profile: 5 min at 95°C, then 37 cycles of 10 s at 95°C and 12 s at 62°C. Quantitative real time PCR (qPCR) assays were analyzed with an MxPro software as described earlier (13). The primers used for PCR are listed in Supplementary Table S3.

### Quantification of SUV39H2 isoforms ratio and statistical analysis

HeLa, MCF7 and SKOV3-ip cells were transfected (RNAiMax, Invitrogen) with 20 nM siRNAs (SIGMA) to deplete splicing factors, and total RNA was extracted at 96 h post-transfection. Specific alternative splicing events were analyzed by RT-PCR, quantified by DNA high sensitive microcapillary CHIP (BioAnalyzer, Agilent). Wilcoxon Signed-Rank test was used to evaluate the significance of effects using two experiments performed in three different cell lines. Because the size of *N* is low (*N* = 6), we have used the *W*-value to evaluate the null hypothesis asserting that the medians of the two conditions are identical. All tests were two-tailed, and statistical significance was considered for *P* < 0.05.

### Western blot, antibodies

Total proteins were separated by electrophoresis on 4–12% gradient PAGE gels (Bio-Rad, #345–0124) and transferred on nitrocellulose membrane (Bio-Rad, #1620115) for western blot. The following antibodies were used for western blot: anti-G9A (Sigma, #HPA050550), anti-SUV39H2 (mix of 1/3 Active Motif, #61449 and 2/3 Proteintech, #11338-AP, (see Supplementary Figure S6), anti-Brg1 (Euromedex, #2SN-2E12-AS), anti-V5 (Life Technologies, #R960–25), anti-Ubiquitin (mAb P4D1, Cell Signalling, #3936), anti-tubulin (Abcam, #ab56676), anti-HP1α (Euromedex, #2HP-2G9-AS), anti-H3 (Abcam, #ab1791), anti-H3K9me2 (Millipore, #07–441), anti-H3K9me3 (Abcam, #ab8898), anti-Rabbit IgG HRP (GE Healthcare, #NA934V), anti-Mouse IgG HRP (Abcam, #ab6808). Western blot signal was acquired with Odyssey Fc system (Licor) and analyzed with the Image Studio software.

### Chromatin ImmunoPrecipitation (ChIP) experiments

The cells were fixed with 1% (v/v) formaldehyde for 10 min at room temperature and the chromatin preparation was processed as described (13). Chromatin was incubated overnight with 1 μg of specific or nonimmune IgG antibodies. Saturated magnetic beads coupled to anti-rabbit antibodies (Dynabeads, Invitrogen, #11204D) were incubated during 2 h to recover the complexes. The beads were washed extensively and nucleic acids were purified and quantified by qPCR.

### Constructs and viral transduction

Various cDNA isoforms of *Tomato, G9A, SUV39H2, SRp20, RBM39* and *TRA2β* were cloned in pLVX-IRES-ZsGreen1 lentiviral vector (Clontech, #632187, see Supplementary Figure S3A) for overexpression in human and mouse cells of a bicistronic transcript encoding for the corresponding protein and the ZsGreen1 protein. Flag and V5 tag sequences were added upstream of each cDNA, and the SV40 NLS sequence was inserted in frame downstream of *Tomato* cDNA. Sequences of all these constructs were checked by sequencing. For the virus production, 293T cells (500 cm^2^ dish) at 50% confluence were transfected with a mix containing 80 μg gag/pol vector (Addgene, # Plasmid 14887), 16 μg pCMV-VSV-G vector (Addgene, #Plasmid 8454), 80 μg pLVX construct and 352 μg of PEI (MW ∼25000; Polysciences Inc., # 23966); medium was changed after 6–8 h and cells were grown for 48 h at 37°C. Medium were filtrated onto 0.45 μm PVDF membrane (Millipore, SCHVU05RE) and virus particle were concentrated 200 times by centrifugation on sucrose cushion (20% sucrose, 10 mM Tris pH 7.5, 100 mM NaCl, 1 mM EDTA) at 28000 rpm (SW 28 rotor) for 1 h 30 min at 4°C. Concentrated viruses were aliquoted and stored at -80°C. Transduction efficiency of each virus in HeLa and 293T cells was estimated by detection of the ZsGreen1 fluorescence and experiments were performed 96 h post-transduction.

### Immunofluorescence and antibodies

Transduced HeLa cells grown on coverslips were fixed 30 min with 3.7% paraformaldehyde (v/v) in phosphate-buffered saline (PBS), then permeabilized 5 min with triton X-100 0.2% (v/v) in PBS. The protein of interest was immuno-detected by incubation for 1 h with anti-V5 (Life Technologies, #R960–25, 1:200 dilution), or anti-H3K9me2 (Millipore, #07–441, 1:1000 dilution), or anti-H3K9me3 (Abcam, #ab8898, 1:1000 dilution), washed with PBS, incubated with Alexa Fluor 488-conjugated goat anti mouse or rabbit IgG antibodies (Life Technologies, #A-11029 or #A-11034, 1:1000 dilution) for 1 h, washed with PBS. The coverslips were mounted onto slides using antifade reagent (Life Technologies, #S36939) and immunofluorescence images were acquired on a Carl Zeiss Axio Observer Z1 microscope equipped with ApoTome module.

### Protein stability assay

For protein stability assay, the proteins of interests were detected after treatment of HeLa cells with 2 μM MG-132 (Merck Millipore, #474790), or with 20 μM of cycloheximide (Sigma, #C-6255) at indicated times. Poly-ubiquitin chains (Ub3–7, K63-linked) used as ubiquitin control (BostonBiochem).

### Cell fractionation

A 60 cm^2^ plate of HeLa cells (90% confluence) was resuspended in 600 μl of B1 buffer (10 mM Tris pH 7.9, 10 mM KCL, 1.5 mM MgCl_2_, 0.34 M sucrose, 10% glycerol) complemented with 0.2% triton X-100 and incubated 5 min on ice. The supernatant F1 was harvested after centrifugation 1000 g, 1 min, 4°C and the packed nuclei washed once with B1 buffer before incubation in 600 μl of B2 Buffer (3 mM EDTA / 0.2 mM EGTA) 30 min on ice. Again the supernatant F2 was collected after centrifugation at (1400 g, 1 min, 4°C), while the pellet was resuspended in 600 μl of B3 buffer (25 mM Tris pH 7.4, 450 mM KCl, 2.5% glycerol, 0.3% NP-40) complemented with 12 μl of Turbo DNase (Applied, #AM1907) and incubated 15 min at 37°C with gentle shaking. Thereafter, the extract was applied to sonication with a Bioruptor (Diagenode; 10 s ON, 15 s OFF, high intensity) and incubated again 15 min at 37°C; this step was repeated twice before centrifugation at 12000 g, 10 min, 4°C to collect the supernatant F3 and insoluble pellet. All buffers were complemented with 0.1 mM DTT, 10 μM PMSF and protease inhibitor cocktail (Roche, #11873580001). The proteins separated by this procedure were analyzed by western blot using a volume of each fraction corresponding to an equal number of cells.

### Recombinant proteins and *in vitro* histone methyltransferase (HMT) assay

For each recombinant protein, two plates (150 cm^2^) of 293T cells were transduced with virus issued from the corresponding pLVX construct. After 48 h, the cells were collected and fractionated as described above. The F1, F2, F3 fractions were pooled together and filtered onto 0.45 μM cellulose acetate membrane (Costar, #8163). Extracts were incubated with 20 μl of packed anti-Flag M2 agarose (Sigma, #A2220) 3 h at 4°C under rotation, then washed two times with HS buffer (25 mM Tris pH 7.4, 450 mM KCl, 2.5% glycerol, 0.3% NP-40) and once with the LS buffer (25 mM Tris pH 7.4, 150 mM KCl, 2.5% glycerol, 0.3% NP-40). Finally, the isolated proteins were eluted twice with 50 μl of 100 μg/ml 3X FLAG peptides (Sigma, #F4799) with shaking, 15 min at room temperature. Purified proteins were stored at -80°C in LS buffer supplemented with 10% glycerol, and their quantity estimated by western blot using anti-V5 antibody. For *in vitro* HMT assay, the purified Flag-V5-tagged proteins and 1 μg of recombinant histone H3.1 (NEB, #M2503S) were incubated in HMT buffer (1 mM of S-adenosylmethionine (NEB, #B9003S), 25 mM Tris pH 8, 10% glycerol) 1 h at 37°C. Then, *in vitro* reaction were analyzed by western blot to follow histone methylation levels as indicated in figure.

### High-throughput RNA sequencing and bioinformatic analysis

HeLa cells were transduced during 96 h with the corresponding viruses, and total RNA extracted as described above. The RNA quality was evaluated by capillary electrophoresis (Agilent 2100 Bioanalyzer system) then cDNA libraries were prepared, split and ligated with adaptators. Single end (1 × 50 bp) sequencing was performed using Illumina HiSeq 2500 technology and each sample was sequenced in triplicate with a minimum of 25 millions sequenced reads per replicate. Details on number of sequenced reads for each sample are given in Supplementary Table S4. The RNA-Seq data analysis was performed by GenoSplice (http://www.genosplice.com). First, reads were aligned onto the human genome (hg19) using Bowtie software (v.0.12.7). Unmapped reads were aligned on an exon–exon junction database built using annotations from version 2013_1 of FAST-DB (see http://www.easana.com). This exon–exon junction database was constituted of known junctions, and putative junctions coming from the combination of all possible exon boundaries. For each gene present in FAST DB v2013_1, reads aligning on constitutive regions (that are not prone to alternative splicing) were counted. Based on these read counts, normalization and differential gene expression were performed using DESeq (v1.12.0 on R v3.0.0). EASANA was used for visualization of results (http://www.easana.com).

## RESULTS

*In silico* analysis of Ref-Seq transcriptome data revealed that a majority of genes encoding chromatin factors contain alternative exons. The inclusion of these exons in mature transcripts is likely to be regulated, potentially leading to produce distinct proteins. The biological role of various versions of chromatin factors that could be generated by alternative splicing is usually not considered in functional studies. Here, we have assayed the functions of the G9A and SUV39H2 protein isoforms. In particular, we focused on the *G9A* cassette exon 10 that can either be skipped (*G9A_Δe10*) or included (*G9A_e10*), and on *SUV39H2* exon 3 that can be skipped (*SUV39H2_Δe3*), fully included (*SUV39H2_e3L*), or partially included (*SUV39H2_e3S*) through the use of a cryptic 5′ splice site (Supplementary Figure S1A).

### Alternatively splicing affects a significant part of *G9A* and *SUV39H2* transcripts

**Figure 1.**
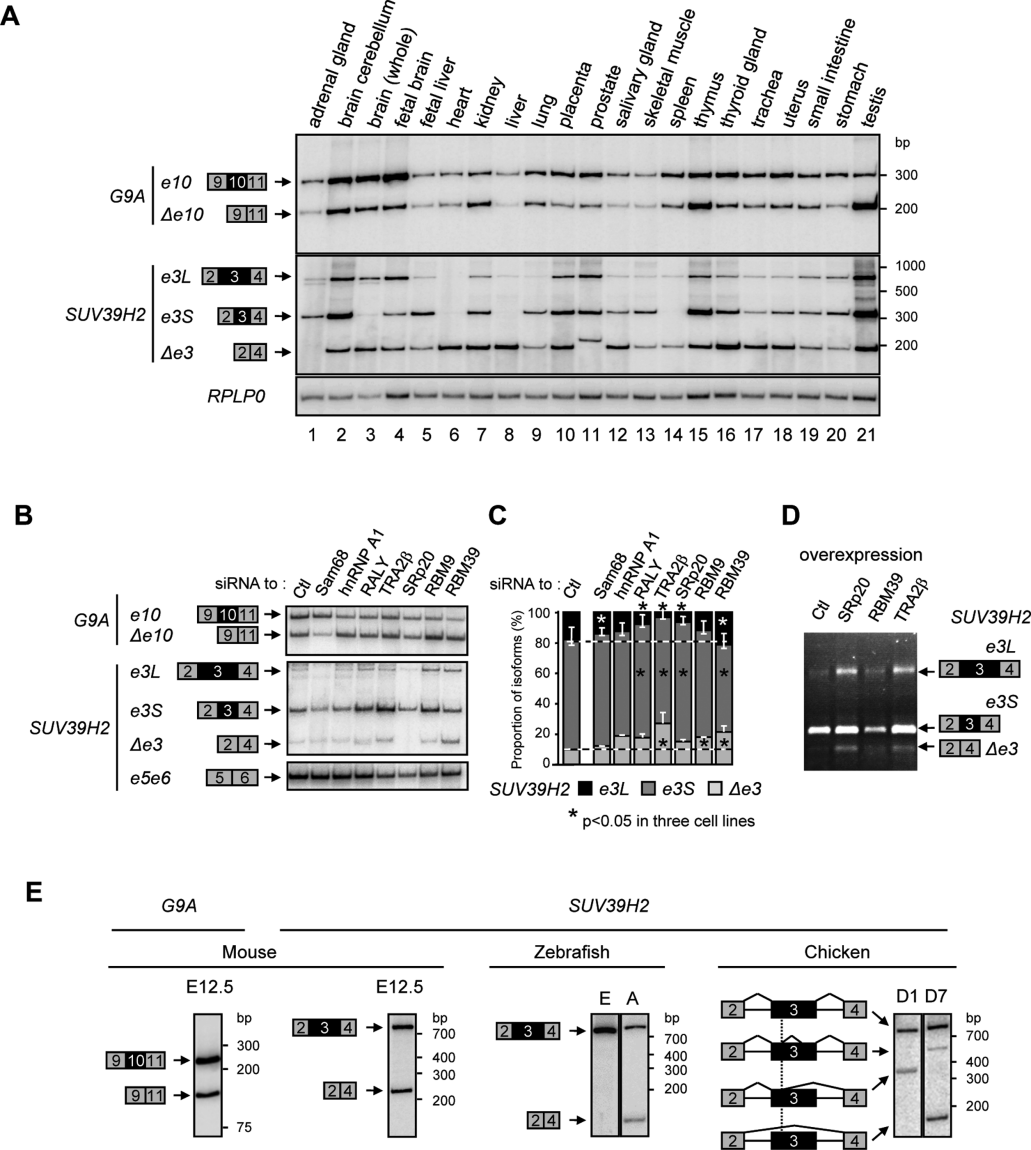
Alternative splicing regulates the expression of *G9A* and *SUV39H2*. (**A**) Analysis in 21 human tissues of inclusion of *G9A* exon 10 (*e10*, exon 10 included; *Δe10*, exon 10 skipped) and *SUV39H2* exon 3 (*e3L*, exon 3 fully included; *e3S*, exon 3 partially included; and *Δe3*, exon 3 skipped). Semi-quantitative RT-PCR was performed on total RNA using radiolabeled primers. PCR products are indicated on the left, and level of *RPLP0* transcripts were used as control. (**B**) Regulation of *G9A* exon 10 and *SUV39H2* exon 3 by splicing factors. The alternative inclusion of *G9A* exon 10 and *SUV39H2* exon 3 was assessed in HeLa cells by siRNA-mediated depletion of indicated factors. Various spliced isoforms were amplified as in the panel A, while SUV39H2 exons 5 and 6 were amplified as control (bottom). (**C**) Ratio of SUV39H2 spliced isoforms in HeLa cells were quantified by capillary electrophoresis. Data display an average of two experiments, and stars indicate significant changes of isoforms in three cell lines (see also Supplementary Figure S1I) estimated by Wilcoxon-ranked test (*P*-value < 0.05). (**D**) Overexpression in HeLa cells of indicated splicing factors affects SUV39H2 exon 3 splicing. SUV39H2 isoforms were amplified by RT-PCR and analyzed in agarose gel stained with ethidium bromide. Splicing patterns displayed a representative example of three experiments. (**E**) Analysis in different organisms of alternative splicing decisions for orthologous *G9A* exon 10 and *SUV39H2* exon 3. Semi-quantitative RT-PCR was performed with radiolabeled primers and total RNA extracted from: E12.5 mouse embryos, Zebrafish embryos (E) and adult (A), chicken embryos at day 1 (D1) and day 7 (D7). For chicken, the various alternative splicing events of *SUV39H2* exon 3 are indicated with sharp lines. Inclusion of orthologous *G9A* exon 10 was only tested in mouse as the structure of its gene is not conserved in other species.

Levels of alternative exon inclusion were evaluated in 21 human tissues. To this end, we first assessed *G9A* and *SUV39H2* expression levels using primers expected to detect constitutive exons (Supplementary Figure S1B). These levels varied from one sample to the other when compared to a housekeeping gene. Interestingly, we found both *G9A* and *SUV39H2* mRNAs to be broadly expressed in human tissues, although SUV39H2 was described as testis-specific in mouse (34). Next, to estimate the regulation of *G9A* exon 10 and *SUV39H2* exon 3, we used primers framing the variant exons. Alternative inclusion of these exons was detected in all tissues, independently of transcript levels, and their inclusion was not always the most frequent event (Figure 1A). For instance, *G9A_Δe10* is preponderant in kidney, thymus and testis, while *SUV39H2_e3L* is almost undetectable in heart, liver, and lung. Some additional cryptic events were detected for *SUV39H2* in adrenal gland, brain and prostate (Figure 1A, lanes 1, 3 and 11). Furthermore, we observed a notable accumulation of *G9A_Δe10* in epithelial cell lines (SW626, Caco2, and HepG2) compared to more transformed cell lines (SKOV3, HeLa, and MON) and mesenchymal cell lines (IMR90 and WI38) (Supplementary Figure S1C, D, E). Altogether, our data suggest that the pool of *G9A* and *SUV39H2* mRNAs is regulated by alternative splicing.

To validate this hypothesis and discriminate between alternative splicing regulation and other sources of transcript heterogeneity (i.e. RNA stability, pseudogene expression), we evaluated the strength of the splice sites at *G9A* exon 10 and *SUV39H2* exon 3, as well as their modulation by splicing regulators. *In silico* predictions did not reveal particular weak splice sites flanking at these exons in comparison to others present in the *G9A* and *SUV39H2* genes (Supplementary Figure S1F). The impact of splicing regulators on the inclusion of the exons was then evaluated by siRNA-mediated depletion of 50 different RNA binding proteins (RBP) on a high-throughput RT-PCR screening platform (38,39). Among these, seven factors were retained regarding their ability to modulate inclusion of one or both alternative exons, and their involvement in the early step of splice site selection (40). This group included hnRNPs (RALY/HNRPCL2; hnRNAP1), SR proteins (SRp20/SRSF3; TRA2β/SRSF10) and RBPs (Sam68/KHDRBS1; RBM9/RBFOX2; RBM39/CAPER). Validation experiments showed that depletions of Sam68, TRA2β, and RBM39 with siRNAs modulate *G9A* exon 10 inclusion in HeLa, MCF7, and SKOV3-ip cells (Figure 1B and Supplementary Figure S1G, H). Significant changes in *SUV39H2* exon 3 inclusion were also observed upon depletion of either Sam68, RALY, TRA2β, SRp20, RBM9, or RBM39 (Figure 1B, C and Supplementary Figure S1G, H). Consistent with this, overexpression of TRA2β, SRp20, or RBM39 in HeLa cells also affect inclusion of this exon (Figure 1D and Supplementary Figure S1J). These data strongly support a main role of splicing for regulating the production of transcripts either containing or lacking *G9A* exon 10 and *SUV39H2* exon 3. Interestingly, inclusion of ortholog exons is also regulated in mouse, zebrafish, and chicken for *SUV39H2*, and in mouse for *G9A* (Figure 1E). The conservation of this regulation suggests that alternative splicing is likely important to control the function of the two genes.

### *G9A* and *SUV39H2* produce several protein isoforms

**Figure 2.**
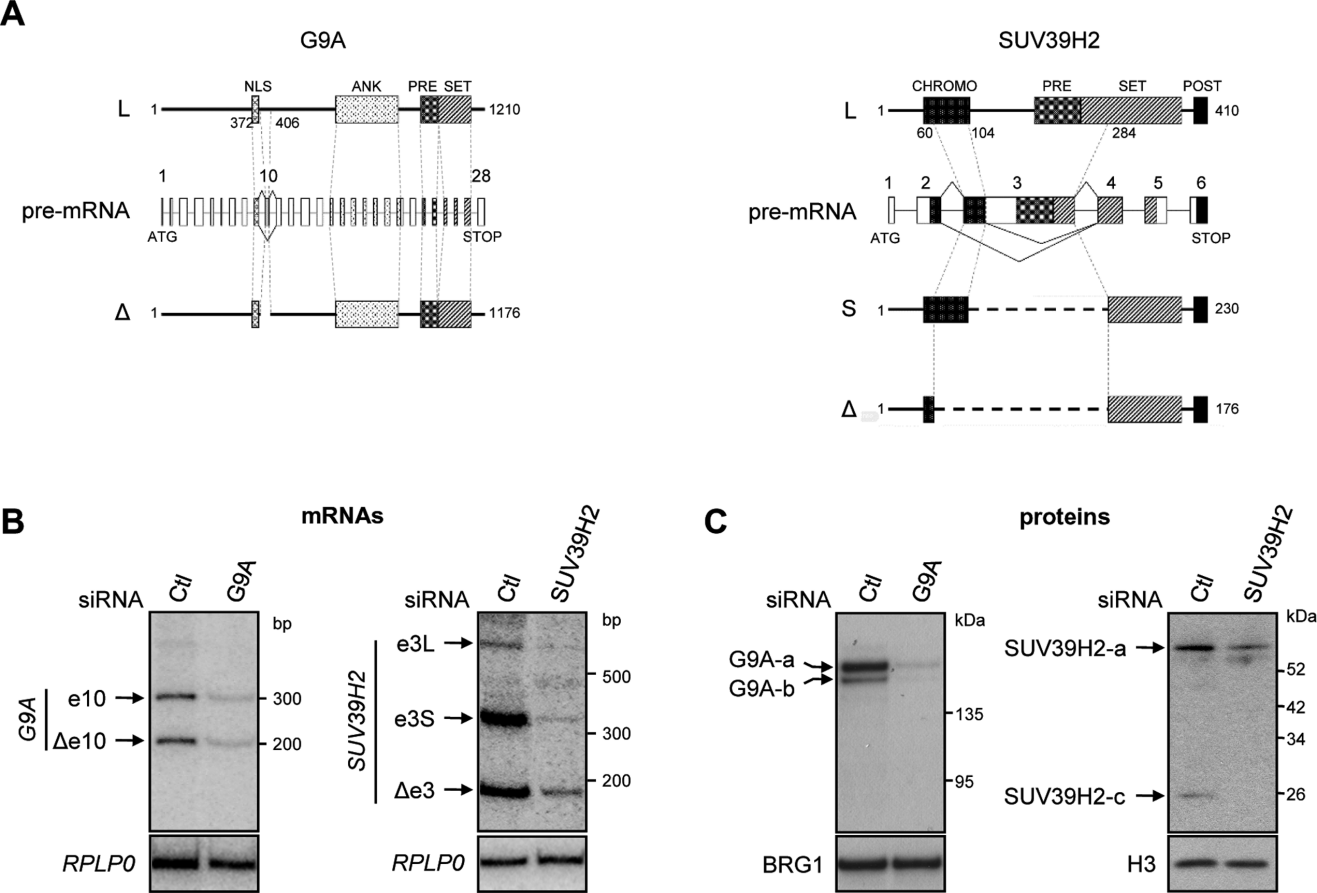
*G9A* and *SUV39H2* produce several protein isoforms. (**A**) Schematic view displaying the protein isoforms encoded by the commonly defined transcript (-L) or versions that artificially reproduced the skipping of *G9A* exon 10 (-Δ) and *SUV39H2* exon 3 (-S and -Δ for partial and total skipping respectively). For pre-mRNA, introns are drawn with bold lines (**—**), exons with boxes, alternative exons of interest with gray boxes, alternative splicing events of pre-mRNA are drawn with sharp lines and the correspondence on protein isoforms are indicated with dotted lines. Known protein domains are reported as follows: nuclear localization signal (NLS), ankyrin (ANK), pre-SET (PRE), SET (SET), post-SET (POST), chromodomain (CHROMO). Symbols and dotted lines were reported on the pre-mRNA to indicate correspondences. (**B**) and (**C**) Analysis in HeLa cells of mRNAs and proteins resulting from *G9A* and *SUV39H2* expression. The specificity of signals detected by radiolabeled RT-PCR (B) or western blot (C) was checked by the analysis of HeLa cells transfected with siRNA: SUV39H2, G9A or control (Ctl). SiRNA were designed to target constitutive exons of *G9A* or *SUV39H2* transcripts. Transcripts levels were assessed 3 days after transfection and proteins levels were detected after 3 days for G9A and 5 days for SUV39H2. *RPLP0* transcripts and BRG1 or H3 proteins were used as controls.

Skipping of *G9A* exon 10 is predicted to generate a protein shorter by 33 amino acids (Figure 2A). The analysis by western blot of various cell lines revealed that anti-G9A antibodies detect two main bands in a total protein extract (noted G9A-a, -b; Supplementary Figure S2A). Interestingly, both signals were separated by a difference in mobility corresponding to 4 kDa, which is consistent with a 33 amino acids deletion. The silencing of G9A expression by siRNA demonstrated the specificity of the western blots (Figure 2B, C, left panels). Furthermore, the ratio between inclusion and skipping of *G9A* exon 10 correlates with the accumulation of each corresponding protein isoform in the different cell lines (compare Supplementary Figure S1C with S2A). These observations suggest that alternative splicing of exon 10 results in the production of two G9A protein isoforms. Regarding *SUV39H2*, skipping or cryptic splicing of exon 3 leads to deletion of 224 or 180 amino acids in the predicted protein isoforms (Figure 2A). As above, a western blot analysis uncovered three bands detected with anti-SUV39H2 antibodies (noted SUV39H2-a, -b and -c; Supplementary Figure S2A). The apparent molecular weight (MW) of the top band (SUV39H2-a) is consistent with a previous analysis of the SUV39H2 protein (34), while two unreported bands (SUV39H2-b and –c) were detected below in the gel. The MW of these bands correspond to these expected from products of alternative splicing of exon 3. The siRNA-mediated depletion of SUV39H2 indicated that the different bands were genuine SUV39H2 products (Figure 2B, C, right panels). Nevertheless, we noted variation in the efficiencies of depletion from one isoform to the other, suggesting differences in the stability of proteins (Supplementary Figure S2B) which would explain the lack of a tight correlation between mRNA and protein ratios (compare Supplementary Figure S1C with S2A). These data sustain that splicing of *G9A* exon 10 and *SUV39H2* exon 3 leads to the production of several protein isoforms.

### The contribution of *G9A* exon 10 and *SUV39H2* exon 3 into their respective protein sequences

G9A and SUV39H2 proteins contain an evolutionary conserved SET domain (*Su(var), E(z)* and *Trithorax*) required for their HMTase activities (Figure 2A). Ankyrin repeats are also present in G9A to mediate protein–protein interactions, while SUV39H2 harbors a chromodomain to bind methylated H3K9. Removal of exon 10 in transcripts does not disturb the organization of G9A protein domains. Conversely, the full and partial skipping of SUV39H2 exon 3 results in a large deletion of the SET domain (SUV39H2-S and SUV39H2-Δ) and in the chromodomain (SUV39H2-Δ).

Such changes in protein could obviously have a direct impact on function of the corresponding factors. To verify this, we designed biscitronic constructs expressing both enzymes and alternative isoforms using the commonly defined cDNA or versions that artificially reproduced the skipping of *G9A* exon 10 and *SUV39H2* exon 3 (Supplementary Figure S3A). We estimate that the expression level of the exogenous factors we produced was approximatively 8-fold higher than that of the corresponding endogenous proteins (Supplementary Figure S3B).

### Alternative inclusion of *SUV39H2* exon 3 is a determinant of protein stability

**Figure 3.**
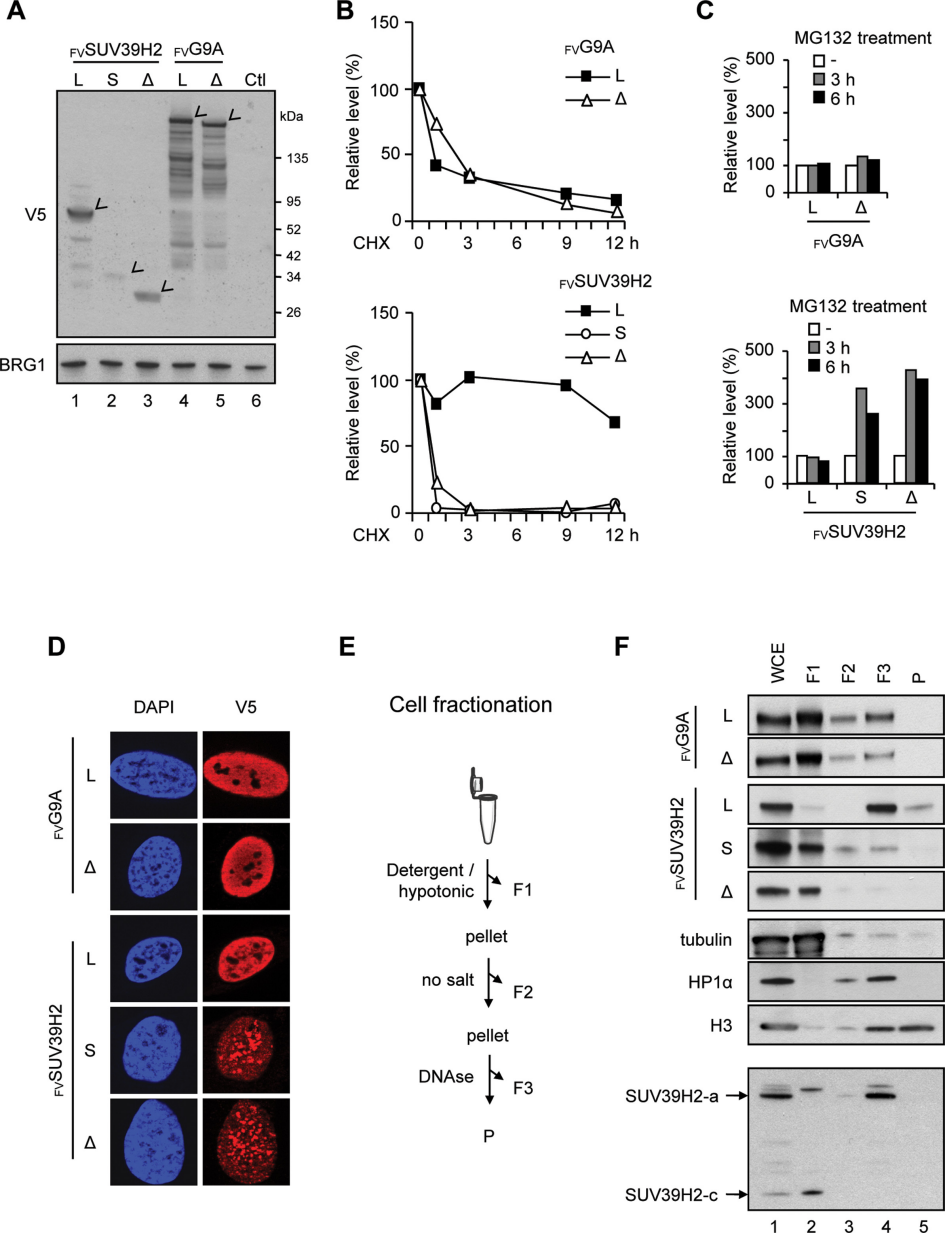
Protein stability and sub-nuclear localization of SUV39H2 isoforms are regulated by alternative inclusion of exon 3. (**A**) Expression in HEK 293T cells of ectopic tagged G9A and SUV39H2 protein isoforms. Cells were transduced with the pLVX vector containing Flag-V5 tags and *SUV39H2* or *G9A* cDNA to express exogenous protein isoforms (_FV_SUV39H2 or _FV_G9A). Cells transduced with pLVX empty vector were used as controls (Ctl). The ectopic proteins were revealed by western blot using the V5 antibody. (**B**) and (**C**) Stability analysis of each G9A and SUV39H2 protein isoform. _FV_G9A and _FV_SUV39H2 isoforms were assessed by western blot in HeLa cells treated with cycloheximide (CHX) (B) or MG132 (C) for the indicated times in hours (h). The signal detected with V5 antibody was normalized to histone H3 levels. (**D**) Localization of _FV_G9A and _FV_SUV39H2 isoforms in HeLa cells. Exogenous proteins were revealed with V5 antibody (red), while DNA was counterstained with DAPI (blue). (**E**) and (**F**) Analysis of _FV_G9A and _FV_SUV39H2 and the endogenous SUV39H2 isoforms after fractionation of HeLa cells. (E) Scheme displaying the cell fractionation procedure. (F) Protein isoforms were detected in whole cell extracts (WCE) and fractions by western blot using the V5 or SUV39H2 antibodies. Endogenous tubulin, HP1α and H3 proteins were analyzed as markers of the cell fractions.

An antibody recognizing the V5 tag revealed in HeLa cells that SUV39H2 protein isoforms accumulated at different levels (Figure 3A). Interestingly, this expression profile was similar between exogenous and endogenous proteins, although the control of their expression was driven by different promoters and gene context (compare Figure 3A, lanes 1 to 3 to Supplementary Figure S2A, lane 6). The differences observed for proteins could not be explained by RNA levels (Supplementary Figure S3C), suggesting that differential protein stability may play an important role in determining the relative abundance of isoforms. To test this possibility, protein synthesis was inhibited in HeLa cells with cycloheximide. This assay revealed a longer half-life for _FV_SUV39H2-L compared to _FV_SUV39H2-S and -Δ, while no difference was detected between _FV_G9A-L and -Δ (Figure 3B, Supplementary Figure S3D). As the proteasome is the principal machinery of protein degradation, we treated HeLa cells with the proteasome inhibitor MG-132. This treatment enhanced detection of _FV_SUV39H2-S and _FV_SUV39H2-Δ, suggesting that these proteins have a high turnover (Figure 3C, Supplementary Figure S3E), although no significant difference in levels of ubiquitination could be detected (Supplementary Figure S3F). We conclude that inclusion of alternative exon 3 is a determinant of protein stability. Moreover, it could provide an explanation for the unequal kinetic of the siRNA-mediated depletion of SUV39H2 proteins (Supplementary Figure S2B).

### Inclusion of exon 3 regulates the sub-nuclear localization of SUV39H2

Sub-cellular localization can influence protein degradation (41). Thus, we investigated the cellular distribution of various G9A and SUV39H2 protein isoforms. In a first approach, we immunolocalized HMTases using the exogenous versions. The _FV_G9A isoforms and _FV_SUV39H2-L showed a nuclear diffused pattern with exclusion from the nucleoli, while shorter versions of SUV39H2 were unexpectedly concentrated in nuclear foci (_FV_SUV39H2-S and -Δ; Figure 3D). To reinforce these results, we performed biochemical fractionation of HeLa cells (Figure 3E, F). In agreement with the data of localization, the two _FV_G9A proteins co-distributed in various fractions, whereas the longest isoform of _FV_SUV39H2 do not co-fractionate with the shortest (compare Figure 3F, lanes 2, 3 to 4, 5). Similar distributions were detected with endogenous SUV39H2 (Figure 3F, bottom panel). The co-distribution of SUV39H2-L with H3 and HP1α suggests that it is more tightly associated with chromatin than the shorter isoforms (Figure 3F, lanes 4 and 5). Furthermore, the tethering of _FV_SUV39H2-L to chromatin may play a role in its higher stability. The differential distribution of SUV39H2 isoforms may reflect their involvement in different complexes. Conversely, the two G9A isoforms may belong to similar complexes. Overall, our results indicate that alternative inclusion of exon 3 defines the sub-cellular localization of SUV39H2.

### Inclusion of exon 3 affects SUV39H2 methyltransferase activity

**Figure 4.**
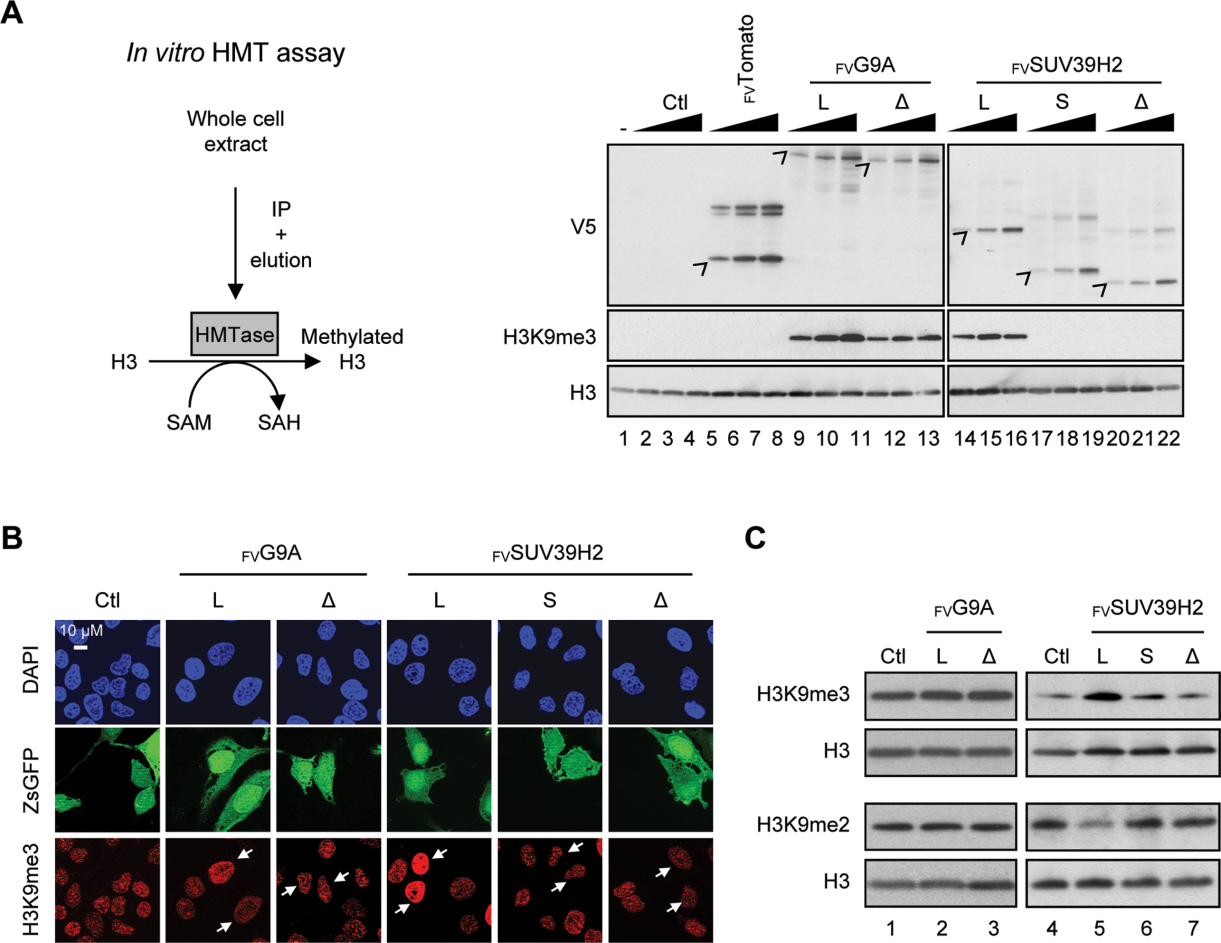
Inclusion of *SUV39H2* exon 3 is required to encode an active histone methyltransferase. (**A**) *In vitro* analysis of the methyltransferase activity associated to each _FV_G9A and _FV_SUV39H2 isoforms. Scheme of the *in vitro* histone methyltransferase (HMT) assay procedure (left panel). Various quantities of purified _FV_G9A and _FV_SUV39H2 (right panel) were incubated with the recombinant human histone H3.1 and S-adénosylméthionine (SAM) producing methylated H3.1 and S-adenosylhomocysteine (SAH). Histone H3 and H3K9me3 were analyzed by western blot using specific antibodies (right panel). Control reactions were supplemented with _FV_Tomato, water (-) or sample issued from blank purification procedure (Ctl). Levels of recombinant proteins were estimated using V5 antibody. (**B**) and (**C**) H3K9me3 was assessed in HeLa cells after expression of _FV_G9A and _FV_SUV39H2 isoforms. (B) DNA was counterstained with DAPI (blue), H3K9me3 was immunostained with a specific antibody (red) and cells expressing _FV_G9A and _FV_SUV39H2 were revealed with ZsGreen1 Fluorescence Protein (ZsGFP panel; labeled with white arrows in H3K9me3 panel). (C) Analysis of H3K9me3, H3K9me2 and H3 levels by western blot in total protein extracts of HeLa cells expressing _FV_G9A and _FV_SUV39H2 isoforms.

We next investigated whether the inclusion of alternative exons influences the activity of G9A and SUV39H2. To this end, isoforms of both enzymes were immunopurified and assayed *in vitro* for their capacity to methylate recombinant histone H3 at the K9 position (Figure 4A). This procedure revealed a strong and specific H3K9 methyltransferase activity of _FV_G9A-L, -Δ and _FV_SUV39H2-L compared to controls performed with water (-), sample issued from purification procedure carried out on untransduced cells (Ctl), and purified _FV_Tomato-NLS (Figure 4A, compare lanes 8–16 with lanes 1–7). As predicted, shorter isoforms of SUV39H2 lacking an intact SET domain were unable to methylate H3K9 (Figure 4A, compare lanes 14–16 with 17–22). The differential activities of SUV39H2 isoforms were corroborated *in vivo* by immunofluorescence microscopy and western blot analysis (Figure 4B; Supplementary Figure S4A, C; Figure 4C). Cells supplemented with _FV_SUV39H2-L showed a drop in the levels of H3K9me2 concomitant with the increase of H3K9me3, suggesting that H3K9me2 is used *in vivo* as a substrate by this enzyme (Figure 4C compare lanes 5 with 4, 6, 7; Supplementary Figure S4B). As for G9A, we did not detect changes in the levels of endogenous H3K9me2/3 after exogenous expression of the two isoforms (see discussion and Figure 4B, C compare lanes 1 with 2, 3; Supplementary Figure S4B). Altogether, our data indicate that the skipping of exon 3 abrogates the H3K9 methyltransferase activity of SUV39H2.

### Alternative inclusion of *SUV39H2* exon 3 modulates the expression of target genes

**Figure 5.**
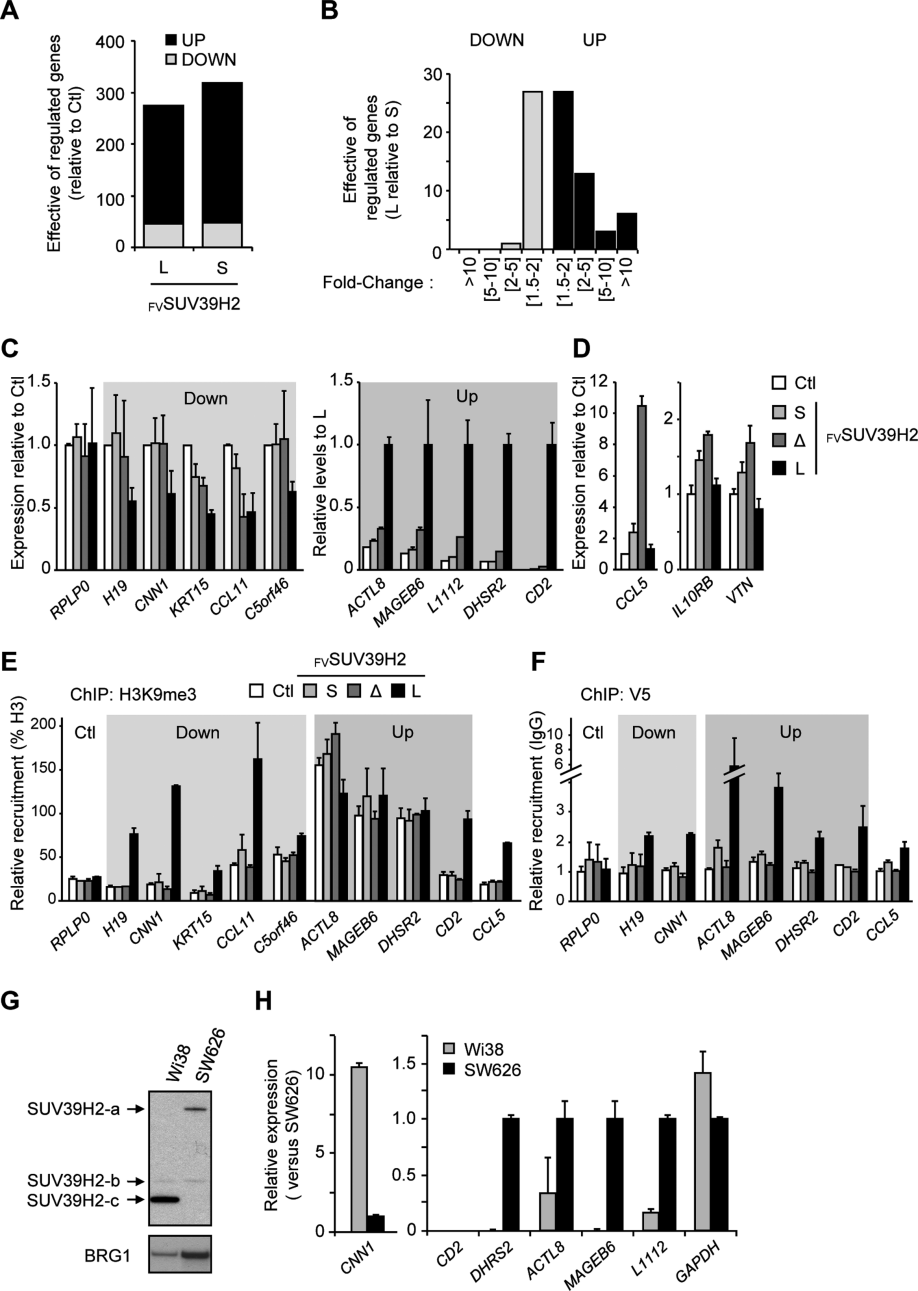
Alternative inclusion of SUV39H2 exon 3 modulates the expression of target genes. (**A**) Graph showing the number of genes that are up- or down-regulated in HeLa cells expressing _FV_SUV39H2-L or _FV_SUV39H2-S. Gene expression levels were assessed by high throughput RNA sequencing and hits were retained with a threshold of 1.5 fold-change (*P*-value < 0.05) when compared to control (Ctl, virus generated with empty construct). (**B**) Graph displaying the differential effect on gene expression of _FV_SUV39H2-L versus _FV_SUV39H2-S. Number of genes is displayed in function of expression fold change. (**C** and **D**) Validation of 13 genes differentially affected in their transcription by SUV39H2 isoforms. Transcription levels were analyzed by RT-qPCR and quantification was displayed as means ± s.e.m. of three experimental replicate. (C) Five genes down-regulated by the over-expression of _FV_SUV39H2-L (Down) were shown relative to control (Ctl). *RPLP0* gene is shown as unaffected gene. Five genes up-regulated by the over-expression of _FV_SUV39H2-L (Up) were presented relative to the expression in presence of _FV_SUV39H2-L (set to 1). (D) Expression analysis of three genes up-regulated specifically by _FV_SUV39H2-S and –Δ isoforms. Transcriptional levels were expressed relative to control (Ctl). (**E** and **F**) Analysis of H3K9me3 and SUV39H2 isoforms at promoters of target genes. Chromatin from cells expressing _FV_SUV39H2 isoforms or not (Ctl) was immunoprecipated with antibodies to H3, H3K9me3, V5 tag or nonimmune (IgG). Relative enrichments were measured by qPCR using primer sets targeting promoters of indicated genes. Values are means ± s.e.m. of three independent experiments. Amounts of H3K9me3 are expressed in percent of H3 (E), and amounts of _FV_SUV39H2 isoforms are expressed relatively to IgG control (F). (**G**) Endogenous SUV39H2 isoforms expressed in WI38 and SW626 cell lines were detected by western blot. (**H**) Relative gene expression in WI38 and SW626 cells of seven hits validated in panel 5C were quantified by RT-qPCR. *CD2* gene expression is not detectable in both cell lines.

Methylation of H3K9 at promoters regulates transcription (28). As we found that inclusion of *SUV39H2* exon 3 globally modulates levels of H3K9me, we assessed whether changing the relative abundance of the different SUV39H2 isoforms would impact gene expression. To that end, we performed high throughput sequencing of mRNAs isolated from HeLa cells expressing exogenous _FV_SUV39H2-L and -S. The two SUV39H2 isoforms broadly up-regulated gene expression relative to the control and, surprisingly, only a few genes were down-regulated (Figure 5A, Supplementary Figure S5A and Table S2). One third of the target genes (134 up- and 11 down-regulated) were commonly modified by both isoforms (Supplementary Figure S5A). Interestingly, comparing transcription in the presence of either _FV_SUV39H2-L or _FV_SUV39H2-S sustained that the two isoforms have different regulatory activities *in vivo* (Figure 5B). Using RT-qPCR, we validated 10 genes specifically down- or up- regulated by _FV_SUV39H2-L (Figure 5C), while three genes were confirmed to be up-regulated by the shorter isoforms _FV_SUV39H2-S and -Δ (Figure 5D). We also performed a kinetic analysis to determine whether observed effects were sustained over time (Supplementary Figure S5B). Specificity of down- and up- regulation of target genes by _FV_SUV39H2-L overexpression was assessed by siRNA-mediated depletion approaches (Supplementary Figures S2B and S5C). In addition, isoform-specific depletion of endogenous transcripts confirmed that the active form of SUV39H2 repressed the expression of *H19, KRT15*, and *C5orf46* (Supplementary Figure S5D and E). All together, our results show that the ratio between the different SUV39H2 isoforms is critical for normal regulation of target gene transcription.

Considering that the short versions of SUV39H2 isoforms are inactive for H3K9me3 methyltransferase activity, we have assessed by chromatin immunoprecipitation (ChIP) assay whether the transcriptional activity of the target gene was correlated with the H3K9me3 levels present at their respective promoters (Figure 5E). Interestingly, the target genes could be classified in two groups depending on their H3K9me3 enrichment in the absence of any overexpressed SUV39H2 isoform (Ctl, white bar). Genes exhibiting a low basal levels of H3K9me3 (*H19, CNN1, KRT15, CCL11, C5orf46, CD2, CCL5*) were subjected to an increase in the level of this modification upon overexpression of _FV_SUV39H2-L; while those initially carrying high levels of H3K9me3 (*ACTL8, MAGEB6, DHSR2*) remained unchanged, likely because their potential methylation sites are already highly modified and may be saturated. In addition, we examined accumulation of exogenous SUV39H2 by ChIP. Our data revealed that promoter of target genes are well occupied by _FV_SUV39H2-L (Figure 5F), strongly suggesting that _FV_SUV39H2-L acts directly on the promoter of target genes. Interestingly, in WI38 and SW626 cells that express extreme ratios of SUV39H2 isoforms expression levels of some target genes matched those observed in the overexpression experiments (Figure 5G, H). Together, these observations suggest that all splice variants of SUV39H2 have a function in transcription regulation.

## DISCUSSION

Here, we report that the exon 10 of *G9A* and exon 3 of *SUV39H2* are alternative spliced exons. Their status as alternatively spliced exons is conserved in different species, tissues and cell lines, suggesting that regulation of these genes at the level of splicing is functionally relevant. Moreover, we show that each of these two genes expresses multiple protein isoforms, and that inclusion of *SUV39H2* exon 3 affects the stability, the sub-nuclear localization, and the HMTase activity of the SUV39H2 protein. Genome-wide expression analysis further revealed that alternative inclusion of SUV39H2 exon 3 modulates the expression of target genes. Our data also suggest that *G9A* alternative exon 10 controls a function that is distinct from its H3K9 methyltransferase activity.

### Studies on chromatin factors need to take alternative protein isoforms into account

The impact of alternative splicing on the activity of chromatin factors is rarely taken into account in functional studies (Supplementary Table S1). Here, we found that exon composition of both *G9A* and *SUV39H2* is affected by a broad range of splicing factors (Figure 1B, Supplementary Figure S1H and I). Consistent with this, a large fraction of endogenous *G9A* and *SUV39H2* transcripts lacks exon 10 and 3, respectively. Furthermore, we found that *SUV39H2* HTMase activity is dependent on the inclusion of exon 3 that is extensively modulated by alternative splicing in 21 human tissues (Figure 1A). Overall, our data reinforce the importance of considering all splice variants when conducting functional studies on chromatin factors.

Interestingly, the expression of SUV39H2 was detected in all tissues, while it was initially described as testis-specific (42). This discrepancy may reflect differences in the procedure used to evaluate gene expression. In fact, the initial study of O'Carroll *et al.* assessed SUV39H2 expression by northern blot using a probe targeting exon 3, which led them to miss transcripts lacking this exon. In agreement with an ubiquitous expression of SUV39H2, Suv39h1/Suv39h2 double null mice exhibit a stronger phenotype than just the Suv39h1 null mice, suggesting that also mouse Suv39h2 has a function beyond that observed in testis (35,43).

We found that the level of alternative SUV39H2 transcripts and proteins were not proportionally related, unlike G9A isoforms. For instance, the SUV39H2_Δe3 transcript is the least abundant in IMR90 and WI38 cells, whereas the most expressed protein isoform is the shortest, likely generated by transcripts lacking exon 3 (Supplementary Figures S1C and S2A). While these observations highlight the role of alternative splicing in generating protein diversity, they also point out its role in regulating protein levels. Moreover, we note that the SUV39H2 isoform encoded by SUV39H2_Δe3 strongly enhances the expression of *CCL5* compared to SUV39H2_e3S and SUV39H2_e3L (Figure 5D and Supplementary Figure S5C). This illustrates that alternative splicing also impacts on transcription through its modulation of various chromatin factor isoforms.

### Inclusion of exon 10 in *G9A* does not affect its H3K9 methyltransferase activity

The most of the abundant literature about the G9A methyltransferase focus only on one cDNA. Therefore, our finding that exon 10 is ubiquitously regulated by alternative splicing and likely translated in protein isoforms appear as an important point (Figures 1A and 2C). The alternative splicing of G9A exon 10 occurs in both human and mouse. Yet, this exon did not influence the properties of G9A as assayed in our study (H3K9 methylation activity, protein stability, sub-nuclear localization). It is possible that sensitivity of the approaches we used to probe G9A activity was insufficient. Although the H3K9 is a preferential substrate for G9A, testing other substrates such as H3K27 or H3K56, or non-histone proteins may be required to reveal isoform-specific activities (29,44–48). Finally, exon 10 is predicted to encode potential sites of phosphorylation, which could be targeted by signaling pathways, and *in fine* regulates G9A activity under specific conditions. For instance, *G9A* could be involved in epithelial-mesenchymal transition (49), and interestingly *G9A* splicing pattern correlates with the epithelial *versus* mesenchymal phenotype (Supplementary Figure S1C, D).

### SUV39H2 and G9A do not use the same pathway to methylate H3K9

*In vitro*, G9A exhibited strong H3K9 methyltransferase activity, while in HeLa cells, its overexpression did not change levels of H3K9me3 and H3K9me2 (Figure 4 and Supplementary Figure S4). This discrepancy between the *in vitro* and *in vivo* activities may indicate that G9A activity is broadly regulated by co-factors in cells. Candidates could be GLP, another HMTase interacting physically and functionally with G9A *in vivo* (50,51). Accordingly, G9A is part of multimeric complexes composed of SUV39H1, GLP, and SETDB1, and has been proposed to participate in the gene silencing activity (50,52). Unlike G9A, the activity of SUV39H2 was detectable both *in vitro* and *in vivo*. Thus, the differences observed between G9A and SUV39H2 activity suggest that these enzymes may not be similarly controlled *in vivo*.

### High levels of SUV39H2 enhances the expression of target genes

The presence of H3K9me3 at promoters is frequently described as a mark of transcriptional repression. Therefore, it was unexpected that increasing level of active SUV39H2 led to a higher number of genes for which transcription was activated rather than repressed in HeLa cells. Such regulation was also observed in cell displaying specifically an endogenous expression of SUV39H2–a (Figure 5G, H). We cannot rule out indirect effects inherent to our technical approaches. Indeed, our results may reflect a competition between the overexpressed HMTase and transcriptional repressors interacting with H3K9, as shown for SUV39H1 (53–55). Nevertheless, for a subset of validated genes, we found a close correlation between the transcription activity and the presence of active SUV39H2 at the promoter (Figure 5E and F), thus SUV39H2 and H3K9me3 might be also involved in transcriptional activation. An activating effect of H3K9me3 is also sustained by other studies, which have found this mark inside the body of transcribed gene (56–58).

### Splicing regulation of chromatin factors extends crosstalk between machineries

Several mechanisms may connect chromatin, transcription and splicing (10,59). To date, most studies have focused on the crosstalk occurring between the different machineries at specific loci. However, their interconnection may also be considered through feedback loops that affect the expression of factors composing these machineries. Our results demonstrate the essential role of alternative splicing in regulating the expression of SUV39H2 and G9A, and this process impacts SUV39H2 functions in transcription. More globally, in accordance with few other studies (Supplementary Table S1), our work highlights the impact of alternative splicing on chromatin properties and transcriptional regulation.

## SUPPLEMENTARY DATA

Supplementary Data are available at NAR Online.

SUPPLEMENTARY DATA
