# Supplementary material for: Alternative splicing regulates the expression of G9A and SUV39H2 methyltransferases, and dramatically changes SUV39H2 functions
Source: Nucleic Acids Res. 2015 Jan 20;43(3):1869–82. doi: 10.1093/nar/gkv013 (PMC4330376; doi:10.1093/nar/gkv013)
Supplement: SUPPLEMENTARY DATA [file supp_43_3_1869__index.html]

Alternative splicing regulates the expression of G9A and SUV39H2 methyltransferases, and dramatically changes SUV39H2 functions — SUPPLEMENTARY DATA 

# Alternative splicing regulates the expression of G9A and SUV39H2 methyltransferases, and dramatically changes SUV39H2 functions

## SUPPLEMENTARY DATA

**Files in this Data Supplement:**

- SUPPLEMENTARY DATA
