# Supplementary material for: Alternative splicing regulates the expression of G9A and SUV39H2 methyltransferases, and dramatically changes SUV39H2 functions
Source: Nucleic Acids Res. 2015 Jan 20;43(3):1869–82. doi: 10.1093/nar/gkv013 (PMC4330376; doi:10.1093/nar/gkv013)
Supplement: SUPPLEMENTARY DATA [file supp_gkv013_nar-00949-a-2014-File007.pdf]

SUPPLEMENTARY MATERIAL AND METHODS

Cell culture

IMR90, Caco-2, HepG2 and SKOV3 (all from ATCC) cells were maintained in Dubelcco's modified Eagle's medium (Gibco, #31966-021) supplemented with 10% (v/v) foetal bovine serum (Thermo Scientific, #SV30160.03) and 100 U/mL penicillin-streptomycin (Gibco, #15140-122). Rhabdoid cells provide by O. Delatre's lab MON (59) were maintained in Roswell Park Memorial Institute medium (Gibco, #61870-010) supplemented with 10% (v/v) foetal bovine serum and 100 U/mL penicillin-streptomycin.

A

G9A

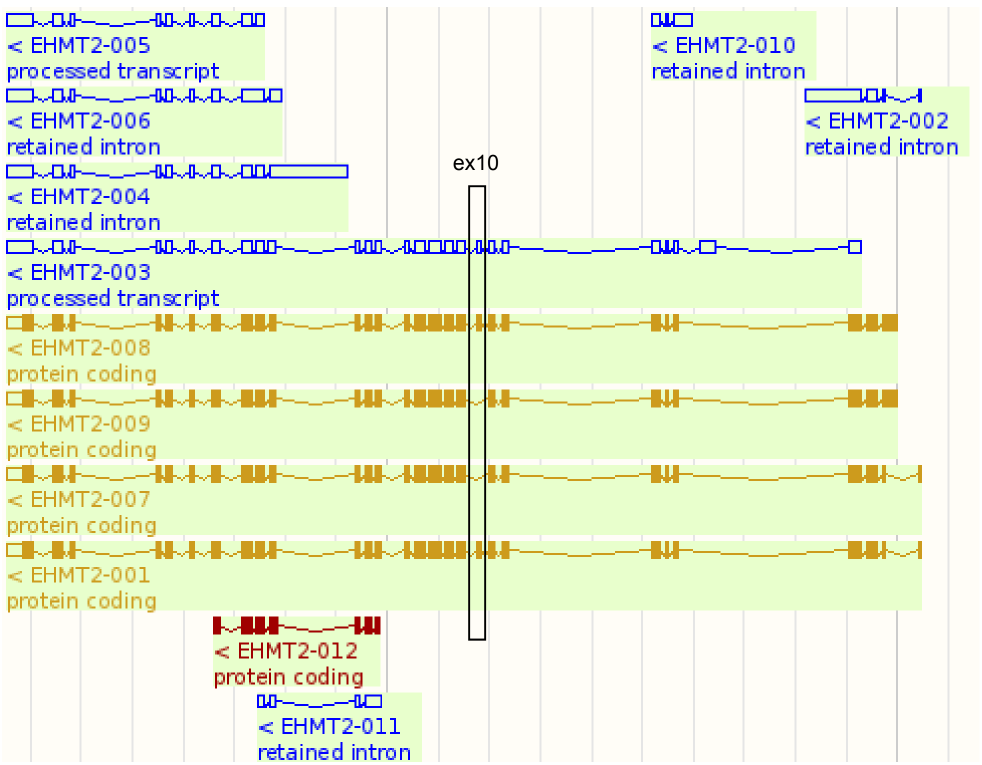

SUV39H2

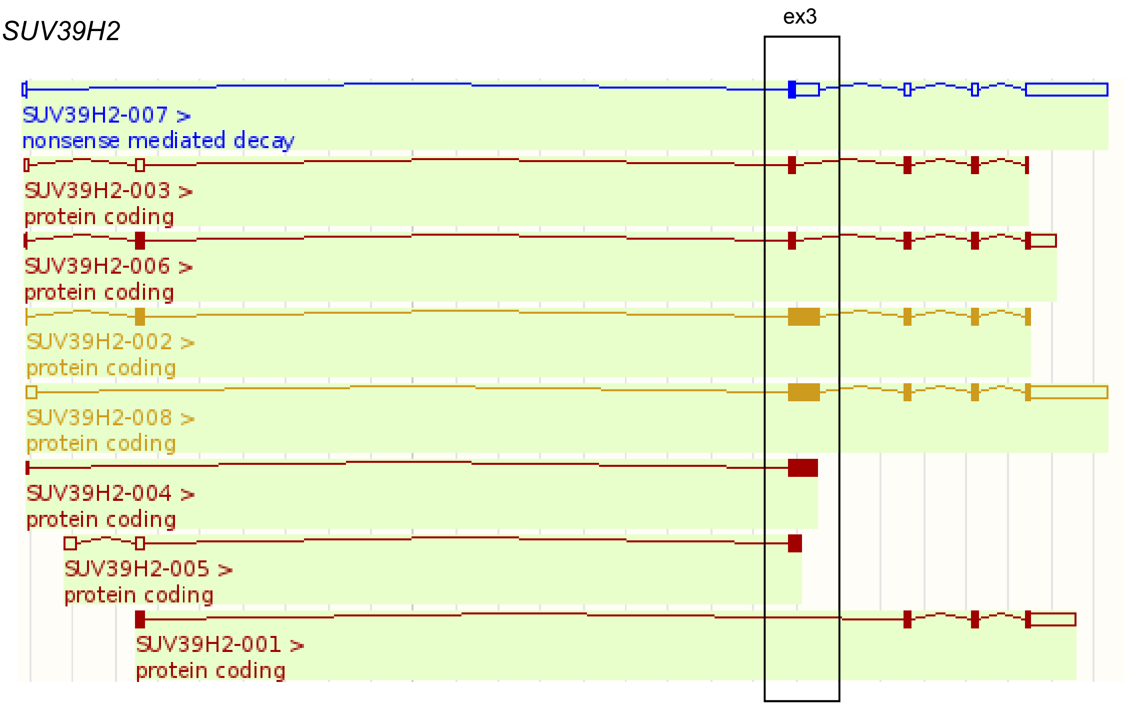

Supplementary Figure S1

Mauger et al.

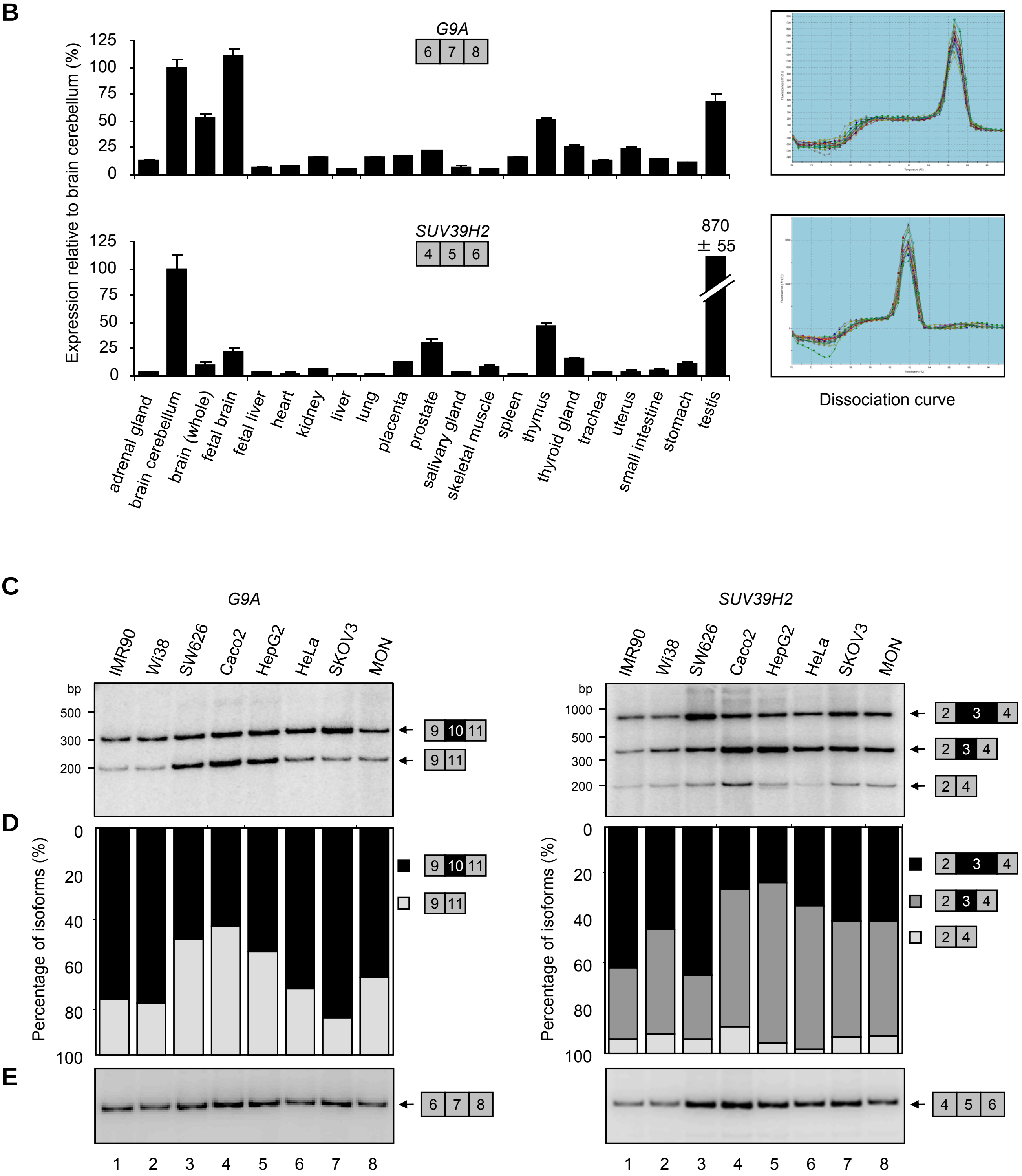

Supplementary Figure S1

Mauger et al.

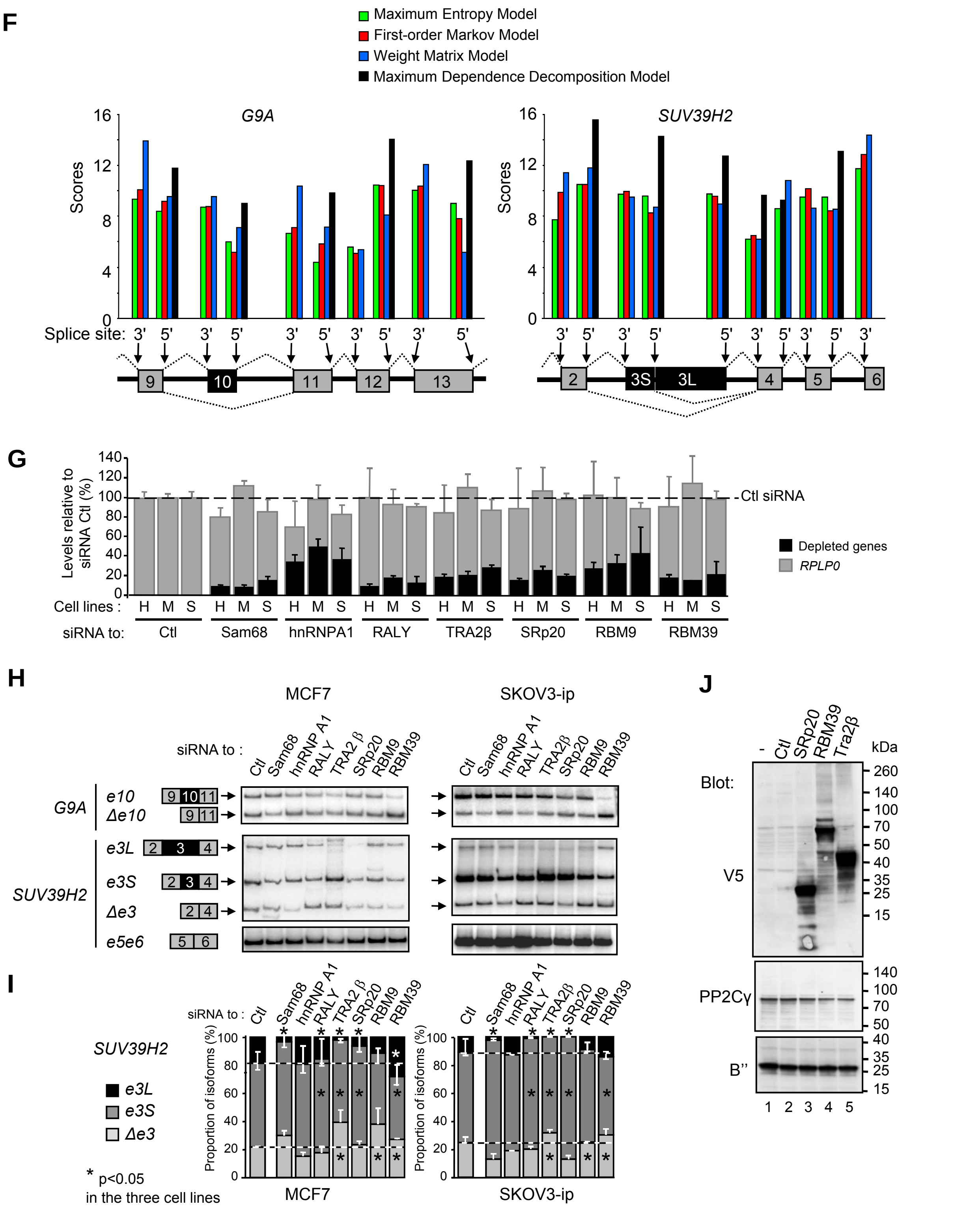

Supplementary Figure S1

Mauger et al.

SUPPLEMENTARY TABLE AND FIGURE LEGENDS

Supplementary Figure S1 related to Figure 1. Alternative splicing regulates the expression of *G9A* and *SUV39H2*. (**A**) Screenshot displaying *G9A* (top) and *SUV39H2* (bottom) transcripts annotated in The Ensembl project (www.ensembl.org). (**B**) Analysis by RT-qPCR of total RNAs from 21 human tissues. The expressions of *G9A* and *SUV39H2* were normalized to cerebellum levels. Dissociation curves of amplicons (right panels) are shown as an evidence of only one PCR product. (**C** and **D**) Analysis of inclusion of *G9A* exon 10 and *SUV39H2* exon 3 in 8 human cell lines. (**C**) Semi-quantitative RT-PCR was performed with radiolabeled primers and total RNA extracted from the different cell lines (pulmonary fibroblasts IMR90 and WI38, ovarian SW626 and SKOV3, colorectal Caco2, hepatic HepG2, cervical HeLa, and rhabdoid MON cells). (**D**) Quantification of each isoform was displayed using 100% stacked histogram bars. (**E**) Total *G9A* and *SUV39H2* expressions were detected by amplification of constitutive exons. (**F**) *In silico* prediction of 5' and 3' splice site strength of G9A and SUV39H2 exons. Indicated models were used from the Burge's lab web site to estimate the strength of splice sites (76). No evidence of particular weakness of alternative exons is observable; for instance, the strength of 3' splice site of G9A exon 12 and SUV39H2 exon 4 are weaker than those calculated for exon 10 and exon 3S, respectively. (**G**) Analysis by RT-qPCR of siRNA-mediated depletion of splicing factor mRNAs was performed in three cell lines: HeLa (H), MCF7 (M) and SKOV3-ip (S). The RNA level of each splicing factor and *RPLP0* was normalized to non targeting siRNA (Ctl). (**H and I**) Analysis of *G9A* exon 10 and *SUV39H2* exon 3 in MCF7 and SKOV3-ip cells after siRNA-mediated depletion of splicing factors. Levels of PCR products were analyzed as in **Figure 1B** and **1C**. (**J**) Overexpression of splicing factors was revealed by Western blot using anti-V5 antibody. PP2Cγ and B" proteins were detected as loading controls.

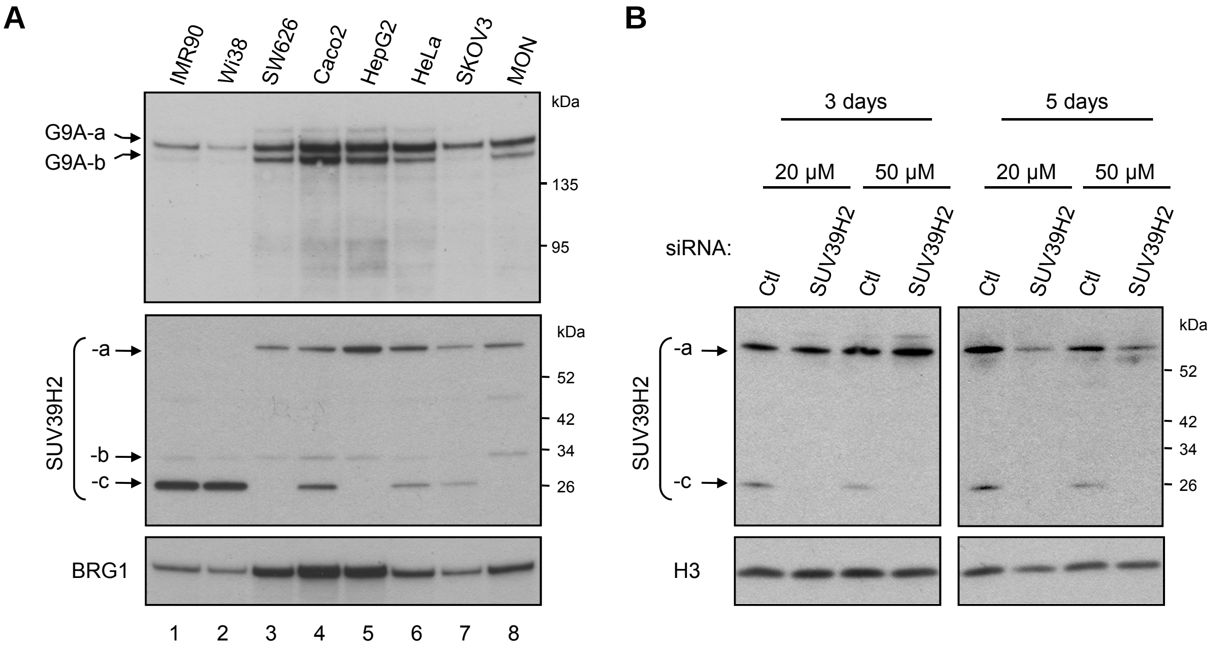

Supplementary Figure S2 related to Figure 2. *G9A* and *SUV39H2* produce several protein isoforms. G9A and SUV39H2 protein isoforms were analysed as in Figure 2C in 8 human cell lines (**A**), after a kinetic of siRNA-mediated depletion for 20 or 50 µM of siRNA (**B**).

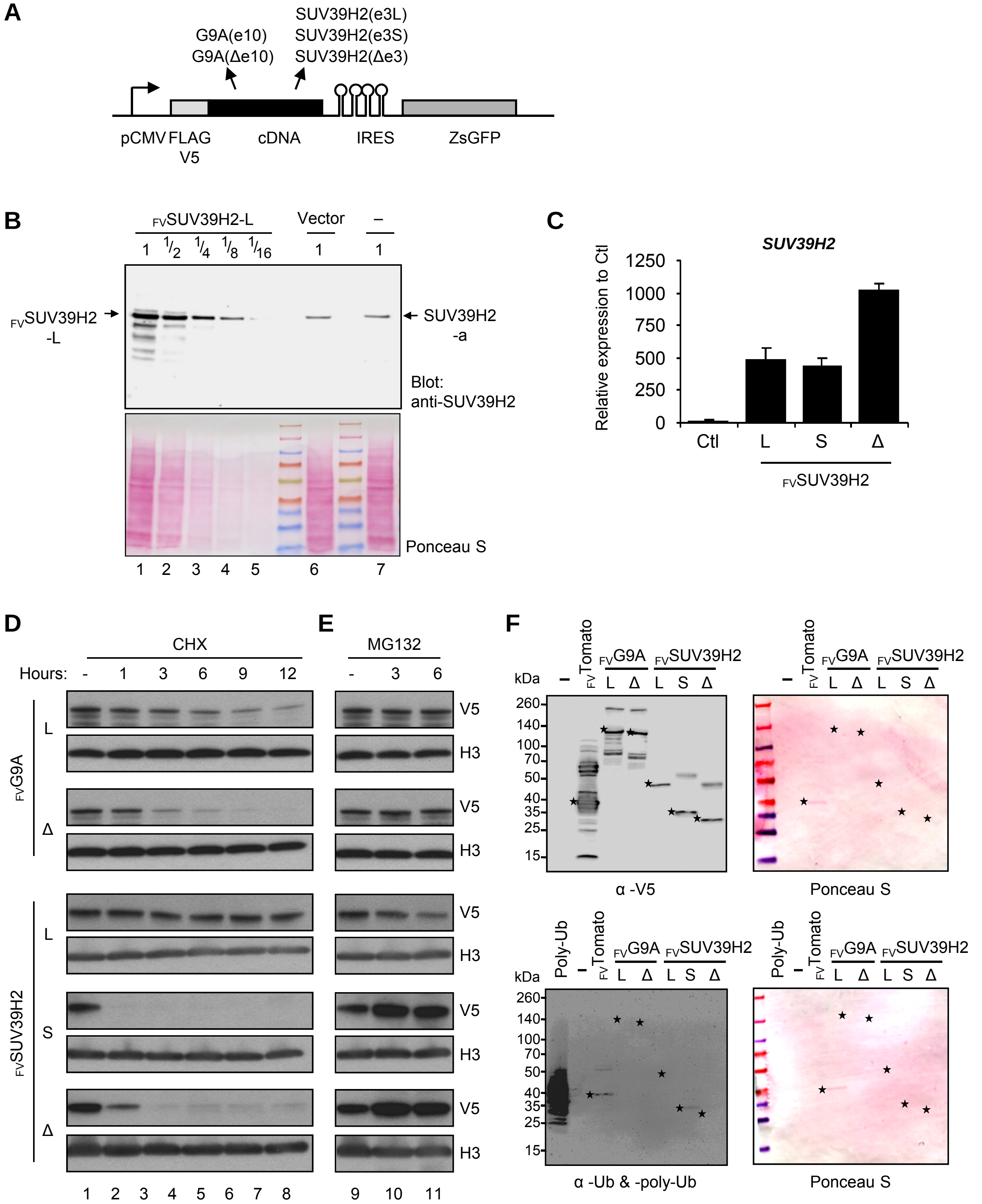

Supplementary Figure S3

Mauger et al.

Supplementary Figure S3 related to Figure 3. Protein stability of SUV39H2 isoforms are regulated by alternative inclusion of exon 3. (**A**) Scheme displaying the biscitronic construct used to express FV*G9A* or FV*SUV39H2* cDNA and ZsGreen1 Fluorescence Protein (ZsGFP) under control of CMV promoter (pCMV). (**B**) Overexpression of FVSUV39H2-L and endogenous SUV39H2 proteins in HeLa cells were revealed by Western blot using anti-SUV39H2 antibody mixture as indicated in material and methods section. Equal quantity of whole protein extracts prepared from Ctl cells (lane 6), untransduced cells (lane 7) or FVSUV39H2-L-expressing cells (lane 1) were compared by assessment of SUV39H2 proteins. The level of over-expression was estimated to 8 fold using serial dilution of FVSUV39H2-L-expressing cell extract (lanes 2-5). (**C**) Levels of *SUV39H2* expression detected RT-qPCR in cells expressing FVSUV39H2-L, -S, or -Δ or transduced with control (Ctl). (**D** and **E**) Analysis of FVG9A and FVSUV39H2 isoforms was assessed by Western blot in HeLa cells untreated (-, lanes 1 and 9) or treated with cycloheximide (CHX) (**D**) or MG132 (**E**) for the indicated times in hours (h). (**F**) Ubiquitination analysis of G9A and SUV39H2 isoforms. Equal quantity of purified G9A and SUV39H2 proteins (as described in **Figure 4A**) were analysed by Western blot using anti-V5 (top) and P4D1 (bottom) antibodies. Corresponding Ponceau staining of the membranes were shown (right panels). The stars indicate the positions of purified proteins as shown in the V5 blot. Positive control for ubiquitin detection was used by co-analysis of Poly-ubiquitin chains (Ub3-7; K63-linked).

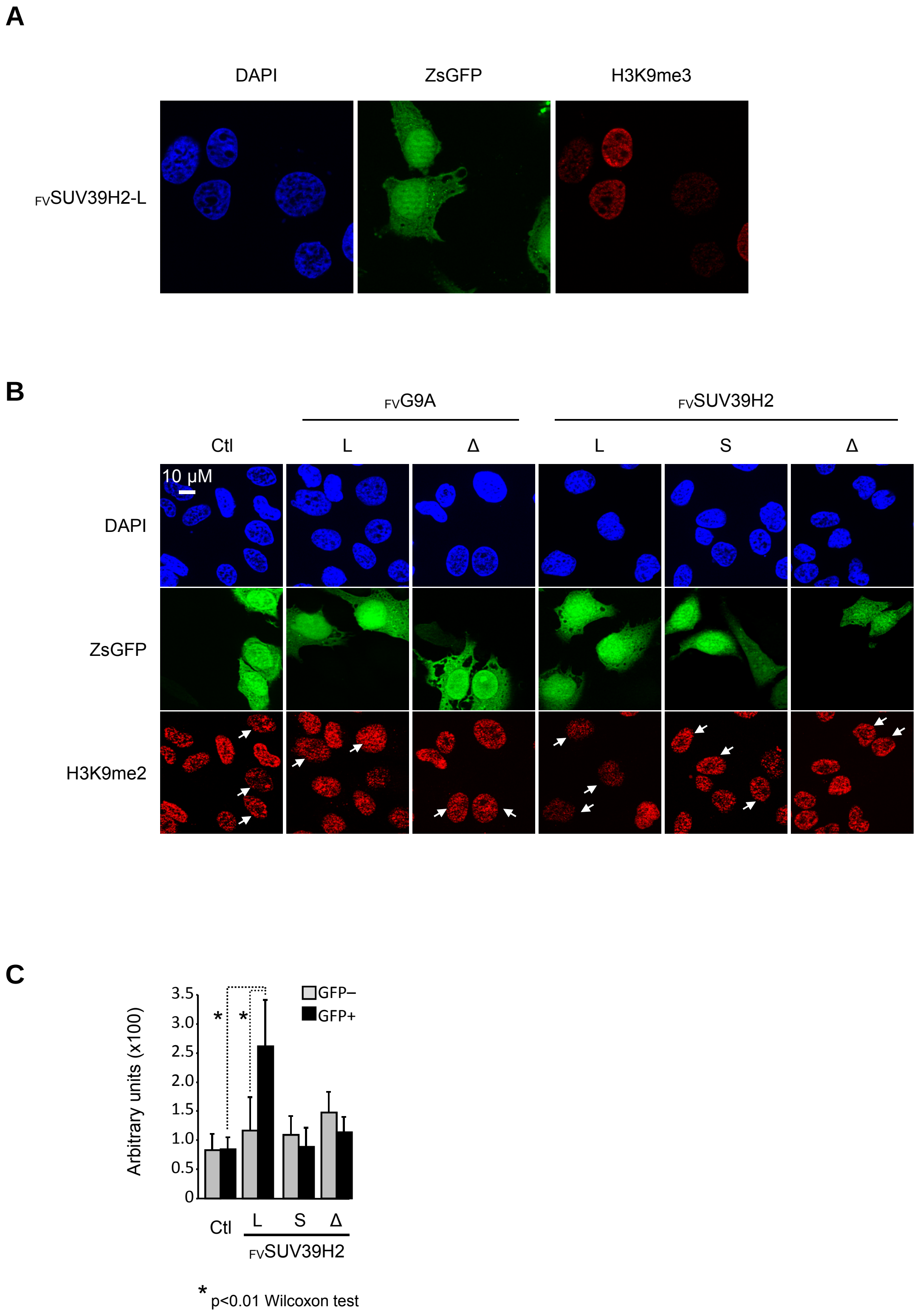

Supplementary Figure S4

Mauger et al.

Supplementary Figure S4 related to Figure 4. Inclusion of *SUV39H2* exon 3 is required to encode an active histone methyltransferase. Immunostaining of H3K9me3 (non saturated picture compared to **Figure 4B**) (**A**) and H3K9me2 (**B**) in HeLa cells expressing $_{FV}$G9A and $_{FV}$SUV39H2 isoforms as in Figure 4.B. (**C**) Quantification of H3K9me3 level detected in imuno-fluorescence assay of **Figure 4B**. Cells over-expressing the $_{FV}$SUV39H2 isoforms were identified by GFP signal (GFP+) compared to control cells (GFP-). Quantification was assessed with duplicate experiments by quantifying at least sixty nuclei of either GFP positive or negative cells in each condition and the significance of differences was established using Wilcoxon non-parametric test ($p<0.01$).

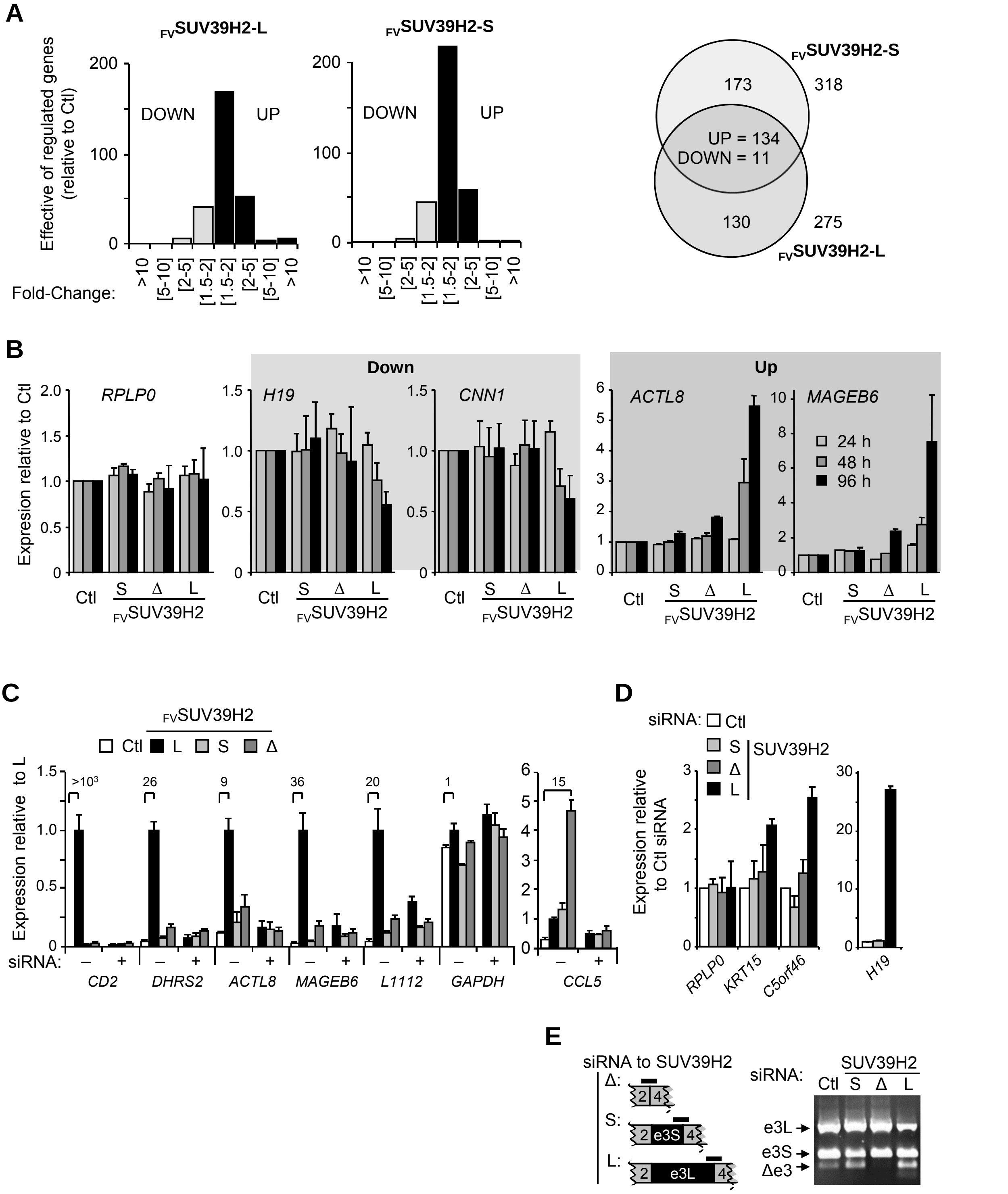

Supplementary Figure S5 Mauger et al.

Supplementary Figure S5 related to Figure 5. Alternative inclusion of SUV39H2 exon 3 modulates the expression of target genes. (**A**) Graph showing the number of genes that are up- or down-regulated in HeLa cells expressing FVSUV39H2-L (left panel) or FVSUV39H2-S (right panel) as described in Figure 5A. Gene numbers are displayed in function of their fold-change. Number of genes regulated specifically and co-regulated by both isoforms are displayed using a Venn diagram (right panel). (**B**) Transcriptional regulation by SUV39H2 isoforms over time. Two genes down-regulated (Down) and two genes up-regulated (Up) by FVSUV39H2-L over-expression were shown relative to control (Ctl). *RPLP0* gene is shown as an unaffected gene. Total RNA extract were performed 1, 2 and 4 days after transduction of exogenous SUV39H2 isoforms. Transcriptional levels of genes were analyzed by RT-qPCR and quantification are displayed as means ± s.e.m. of three experimental replicate. (**C**) Validation by RT-qPCR of 6 hits differentially regulated by SUV39H2 isoforms. The significance of this validation was assessed comparing results obtained in HeLa cells transfected (+) or not (-) by siRNA targeting all transcripts of *SUV39H2*. *GAPDH* gene is shown as unaffected gene. (**D**) Release of repressive effect associated to SUV39H2 using isoform-specific depletion of endogenous transcripts. *RPLP0* gene is shown as unaffected gene. (**E**) RNA analysis of SUV39H2 isoform-specific depletion. Target sequences of isoforms-specific siRNAs were symbolised by black bars under alternatively spliced junctions. SUV39H2 isoforms were amplified by RT-PCR and analysed in agarose gel stained with ethidium bromide. Splicing patterns displayed a representative example of three experiments.

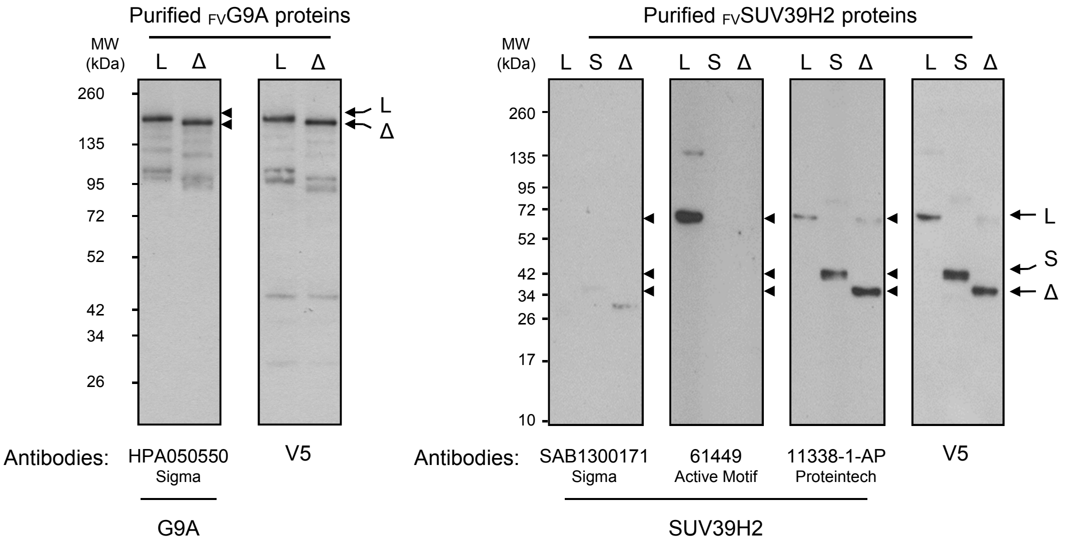

Supplementary Figure S6. Detection by western blot of purified FVG9A and FVSUV39H2 using various antibodies. Efficiency of antibodies was assessed comparing signals obtained with G9A, SUV39H2 or V5 antibodies.

Chromatin regulator (alter. name)	Functions of the main product	isoforms	Functions of the alternative splicing isoforms	Refs
DNMT1	DNA methylation	4 isoforms<br>DNMT1o/s/p specific to oocyte, somatic or spermatocye<br>DNMT1b (exon4)	Localization, translational regulation, sex-specific expression<br><br>exon4 : similar kinetic parameters on DNA methylation	(60, 61)
DNMT3B	DNA methylation	40 isoforms<br>DNMT3B4 B5 lacking exons21+22<br>DNMT3B3-like splice variant lacking exon 5	Exons21+22 encoding catalytic domain<br><br>Exon5 increases DNA binding or influences the 3D structure of the adjacent PWWP domain	(62–64)
CARM1 (PRMT4)	Arginine methylase<br><br>H3R8	4 isoforms in human, mouse & rat<br><br>2 isoforms in *X leavis*	CARM1-exon v3 regulates alternative splicing of CD44 and E1a<br>Exon15 encode an automethylated domain of the protein	(65–67)
SIRT1	NAD-dependent deacetylase<br><br>H4-K16 and H3-K9 deacetylation	SIRT1-ΔExon8<br><br>SIRT1-Δexon2-9	SIRT1- Δ Exon8, displays significant differences in stress-sensitivity, RNA/protein stability, protein-protein interactions and deacetylase activity<br>-Δexon2-9 lacks catalytic domain	(68–70)
PKM1/2	Pyruvate kinase (Warburg effect)	2 isoforms : PKM1 and PKM2	PKM2 phosphorylates H3T11p	(71)
LSD1	H3K4 demethylase	4 isoforms	4 amino acid exon E8a brings the phosphorylated Threonin important for protein conformation and activity	(72, 73)
NURF301	Subunit of chromatin remodeling complex	3 isoforms	Truncated C-terminal isoforms lack ability to target H3K4me3 and H4K16Ac	(74)
EZH2	HMTase of H3K27	2 isoforms<br>EZH2α<br>EZH2β brings Exon4 skipping and Exon8 3'SS	EZH2β represses a predominantly unique subset of gene targets from EZH2α	(75)

Supplementary Table S1: Few examples of chromatin regulators functionally studied in toward their alternative spliced isoforms

SUV39H2_S versus SUV39H2_L

FAST DB STABLE ID	Gene Symbol	Regulation	Fold-Change	P-Value	Gene Coordinates (hg19)	Gene Name	Aliases and Synonyms	Entrez Gene ID	EnsEMBL ID	Intensity SUV39H2_L	Intensity SUV39H2_S
GSHG0036106	MIRLET7I	up	2.92	8.29E-03	chr12(+):62997466-62997550	microRNA let-7i	hsa-let-7i	406891	ENSG000001	4.46	13.05
GSHG0031492	INE1	up	1.97	2.16E-02	chrX(+):47064247-47065260	inactivation escape 1 (non-protein coding)	NCRNA00010	8552	ENSG000002	13.94	27.40
GSHG0018727	NNAT	up	1.93	7.28E-03	chr20(+):36149607-36152088	neuronatin	Peg5	4826	ENSG000000	15.45	29.85
GSHG0014590	CNN1	up	1.90	5.90E-07	chr19(+):11649579-11661139	calponin 1, basic, smooth muscle	Sm-Calp, MCC	1264	ENSG000001	51.95	98.69
GSHG0023789	--	up	1.84	2.59E-02	chr5(+):10231010-10231330	--	--	--	--	15.05	27.67
GSHG0013280	CCL5	up	1.81	4.37E-04	chr17(-):34198498-34207377	chemokine (C-C motif) ligand 5	MGC17164, ANTES	6352	ENSG000001	38.14	69.16
GSHG0021365	--	up	1.69	4.56E-02	chr3(+):183165396-183173798	--	--	--	--	17.08	28.85
GSHG0025159	C5orf46	up	1.68	3.37E-03	chr5(-):147269579-147286101	chromosome 5 open reading frame 46	MGC23985	389336	ENSG000001	24.05	40.40
GSHG0010003	ISLR	up	1.67	9.04E-05	chr15(+):74466025-74469213	immunoglobulin superfamily containing leucir	HsT17563	3671	ENSG000001	49.41	82.73
GSHG0020267	LRP5L	up	1.67	2.41E-02	chr22(-):25747386-25777546	low density lipoprotein receptor-related prote	DKFZp434O0213	91355	ENSG000001	23.44	39.21
GSHG0011038	--	up	1.65	1.32E-02	chr16(+):23579994-23580875	--	--	--	--	22.68	37.45
GSHG0025255	KCNMB1	up	1.63	9.16E-04	chr5(-):169805167-169816659	potassium large conductance calcium-activat	hslo-beta	3779	ENSG000001	44.50	72.50
GSHG0012400	CCL11	up	1.62	5.85E-03	chr17(+):32612687-32615199	chemokine (C-C motif) ligand 11	eotaxin, GC22554	6356	ENSG000001	34.48	55.92
GSHG0013210	VTN	up	1.60	6.58E-05	chr17(-):26694298-26697373	vitronectin	VN	7448	ENSG000002	97.40	155.98
GSHG0024540	--	up	1.60	8.22E-03	chr5(+):179286013-179288280	--	--	--	--	33.00	52.81
GSHG0013393	KRT15	up	1.59	5.71E-03	chr17(-):39669998-39678665	keratin 15	CK15, 15, 1CO	3866	ENSG000001	41.66	66.15
GSHG0000343	--	up	1.56	4.89E-02	chr1(+):32706183-32707111	--	--	--	--	23.12	36.16
GSHG0029917	LY6D	up	1.55	2.54E-02	chr8(-):143866298-143868008	lymphocyte antigen 6 complex, locus D	E48	8581	ENSG000001	42.89	66.49
GSHG0022884	GUSBP5	up	1.55	3.42E-02	chr4(+):144480621-144489313	glucuronidase, beta pseudogene 5	--	441046	ENSG000002	28.48	44.12
GSHG0033447	--	up	1.55	1.76E-02	chr6(-):88875078-88876743	--	--	--	--	39.96	61.75
GSHG0026126	--	up	1.54	2.22E-02	chr6(+):151713536-151715281	--	--	--	--	26.77	41.32
GSHG0029908	TSNARE1	up	1.54	1.97E-02	chr8(-):143293442-143484543	t-SNARE domain containing 1	FLJ31164	203062	ENSG000001	34.62	53.32
GSHG0019332	HELZ2	up	1.54	1.41E-02	chr20(-):62189439-62205596	helicase with zinc finger 2, transcriptional coa	KIAA1769, DIP1, RI	85441	ENSG000001	38.04	58.50
GSHG0004938	RARRES3	up	1.53	1.13E-03	chr11(+):63304273-63313929	retinoic acid receptor responder (tazarotene i	HRASLS4, IG3	5920	ENSG000001	71.37	109.12
GSHG0024271	EGR1	up	1.51	5.61E-07	chr5(+):137801169-137805189	early growth response 1	AT225, 0S30, ROX-	1958	ENSG000001	598.26	904.81
GSHG0015033	IGFL1	up	1.51	4.01E-02	chr19(+):46733009-46734500	IGF-like family member 1	UNQ644	374918	ENSG000001	37.65	56.93
GSHG0039495	CBX3P2	up	1.51	2.74E-02	chr18(-):2652169-2655394	chromobox homolog 3 pseudogene 2	--	645158	ENSG000002	24.40	36.88
GSHG0021739	ZMYND10	up	1.51	4.21E-02	chr3(-):50378537-50384283	zinc finger, MYND-type containing 10	BLU	51364	ENSG000000	34.14	51.44
GSHG0005466	H19	up	1.21	9.46E-02	chr11(-):2016406-2019072	H19, imprinted maternally expressed transcri	--	283120	ENSG000001	6.91	7.18
GSHG0000845	CD2	down	Inf	2.50E-10	chr1(+):117297057-117311850	CD2 molecule	--	914	ENSG000001	23.64	0.00
GSHG0025320	AACSP1	down	43.68	1.91E-09	chr5(-):178191862-178245436	acetoacetyl-CoA synthetase pseudogene 1	--	729522	ENSG000002	25.25	0.58
GSHG0031438	MAGEB6	down	21.71	4.16E-35	chrX(+):26210557-26213765	melanoma antigen family B, 6	CT3.4, LJ40242, AG	158809	ENSG000001	84.51	3.89
GSHG0007126	--	down	13.53	7.74E-10	chr12(+):129594222-129597839	RP11-669N7.2	LOC283352	283352	ENSG000002	19.05	1.41
GSHG0000193	ACTL8	down	12.94	1.63E-24	chr1(+):18081808-18153556	actin-like 8	CT57	81569	ENSG000001	118.33	9.14
GSHG0008734	DHRS2	down	11.23	2.17E-21	chr14(+):24099324-24114846	dehydrogenase/reductase (SDR family) mem	HEP27, DR25C1	10202	ENSG000001	71.41	6.36
GSHG0029029	L1112	down	7.87	8.62E-25	chr8(+):58890917-58896683	RP11-1112C15.1	Linc1112		ENSG000002	104.42	13.27
GSHG0005810	TCN1	down	7.08	1.14E-24	chr11(-):59620282-59634041	transcobalamin I (vitamin B12 binding protein	TC1, CI	6947	ENSG000001	104.62	14.77
GSHG0024786	--	down	5.24	4.49E-03	chr5(-):54162400-54162890	--	--	--	--	10.15	1.94
GSHG0023231	COX7B2	down	4.83	2.19E-04	chr4(-):46736840-46911252	cytochrome c oxidase subunit VIIb2	--	170712	ENSG000001	14.78	3.06
GSHG0032096	PAGE1	down	3.19	1.97E-05	chrX(-):49452056-49460596	P antigen family, member 1 (prostate associa	CT16.3, AGE-9, AG	8712	ENSG000000	34.89	10.93
GSHG0003216	SUV39H2	down	3.02	2.22E-103	chr10(+):14920782-14946302	suppressor of variegation 3-9 homolog 2 (Dr	FLJ23414, MT1B	79723	ENSG000001	93338.64	30907.29
GSHG0029002	C8orf22	down	2.75	2.28E-03	chr8(+):49966895-49988642	chromosome 8 open reading frame 22	--	492307	ENSG000001	30.03	10.91
GSHG0040123	LINC00665	down	2.55	1.41E-10	chr19(-):36803961-36822620	long intergenic non-protein coding RNA 665	--	100506930	ENSG000002	111.99	43.93
GSHG0035368	SNORD15B	down	2.50	2.70E-02	chr11(+):75115465-75115610	small nucleolar RNA, C/D box 15B	U15B	114599	ENSG000002	12.40	4.95
GSHG0016158	ZNF772	down	2.45	1.75E-02	chr19(-):57980954-57988938	zinc finger protein 772	DKFZp686I1569	400720	ENSG000001	15.31	6.26
GSHG0031893	CSAG1	down	2.35	2.54E-04	chrX(+):151903228-151909518	chondrosarcoma associated gene 1	CSAGE, T24.1	158511	ENSG000001	41.69	17.74
GSHG0027619	--	down	2.17	3.02E-04	chr7(+):102613959-102629302	--	--	--	--	48.73	22.45
GSHG0021423	TP63	down	2.17	3.09E-04	chr3(+):189349216-189615068	tumor protein p63	EEC3, ET, BP, FC8	8626	ENSG000000	40.51	18.69
GSHG0031252	--	down	2.15	2.17E-02	chr9(-):137711262-137764468	--	--	--	--	19.12	8.87
GSHG0031214	FIBCD1	down	2.14	2.01E-06	chr9(-):133777825-133814455	fibrinogen C domain containing 1	FLJ14810	84929	ENSG000001	100.95	47.18
GSHG0023034	--	down	2.01	2.64E-03	chr4(+):189321890-189523062	--	--	--	--	41.60	20.71
GSHG0022405	FGFR3	down	1.98	4.77E-02	chr4(+):1795034-1810599	fibroblast growth factor receptor 3	CD333, EK2, TK4	2261	ENSG000000	36.96	18.65
GSHG0014861	--	down	1.90	2.94E-03	chr19(+):36980529-36981942	--	--	--	--	39.80	20.94
GSHG0007412	VDR	down	1.90	2.53E-07	chr12(-):48235322-48336934	vitamin D (1, 5- dihydroxyvitamin D3) recepto	NR1I1	7421	ENSG000001	119.68	63.02
GSHG0020652	RARB	down	1.86	4.09E-02	chr3(+):25215823-25639420	retinoic acid receptor, beta	HAP, R1B2, RB2	5915	ENSG000000	19.90	10.72
GSHG0010729	--	down	1.83	1.83E-09	chr15(-):91837333-91838648	--	--	--	--	169.03	92.38
GSHG0022297	ETV5	down	1.75	4.64E-05	chr3(-):185764106-185826901	ets variant 5	ERM	2119	ENSG000002	99.05	56.61
GSHG0003375	DKK1	down	1.74	1.52E-04	chr10(+):54074012-54077416	dickkopf 1 homolog (Xenopus laevis)	DKK-1, K	22943	ENSG000001	95.93	55.06
GSHG0023928	FST	down	1.73	8.03E-04	chr5(+):52776264-52782304	follistatin	FS	10468	ENSG000001	62.10	35.95
GSHG0013836	NOTUM	down	1.72	3.87E-03	chr17(-):79910397-79921512	notum pectinacetylesterase homolog (Drosop	--	147111	ENSG000001	79.63	46.34
GSHG0026305	ELOVL2	down	1.71	2.60E-05	chr6(-):10980988-11044624	ELOVL fatty acid elongase 2	Ssc2	54898	ENSG000001	94.76	55.30
GSHG0008914	HSPA2	down	1.68	3.64E-09	chr14(+):65002623-65009952	heat shock 70kDa protein 2	--	3306	ENSG000001	256.36	152.85
GSHG0015899	ZNF229	down	1.67	5.32E-09	chr19(-):44930426-44952768	zinc finger protein 229	--	7772	ENSG000001	224.23	133.89
GSHG0031668	FAM133A	down	1.66	4.79E-07	chrX(+):92929012-92967273	family with sequence similarity 133, member	CT115, LJ37659, P1	286499	ENSG000001	347.20	208.88
GSHG0028479	CCDC71L	down	1.63	9.91E-08	chr7(-):106299763-106301442	coiled-coil domain containing 71-like	FLJ36031	168455	ENSG000002	223.80	137.02
GSHG0001444	KCNK2	down	1.61	9.54E-05	chr1(+):215178885-215410436	potassium channel, subfamily K, member 2	K2p2.1, REK-1	3776	ENSG000000	144.44	89.91
GSHG0045802	CAMK2N1	down	1.58	9.31E-06	chr1(-):20808885-20812728	calcium/calmodulin-dependent protein kinase	CaMKIINalpha	55450	ENSG000001	183.56	115.92
GSHG0022555	UCHL1	down	1.57	6.73E-09	chr4(+):41258898-41270445	ubiquitin carboxyl-terminal esterase L1 (ubiq	PGP9.5, ch-L1	7345	ENSG000001	342.02	217.40
GSHG0009182	TMEM121	down	1.57	1.16E-02	chr14(+):105992953-105996539	transmembrane protein 121	hole, GC4659	80757	ENSG000001	53.53	34.03
GSHG0026645	NFKBIE	down	1.57	7.24E-03	chr6(-):44225903-44233531	nuclear factor of kappa light polypeptide gen	IKBE	4794	ENSG000001	56.41	36.03
GSHG0005864	RCOR2	down	1.56	2.58E-02	chr11(-):63678694-63684659	REST corepressor 2	--	283248	ENSG000001	45.55	29.11
GSHG0009996	NEO1	down	1.55	4.38E-03	chr15(+):73344825-73597547	neogenin 1	HsT17534, GDCC2,	4756	ENSG000000	129.81	83.58
GSHG0017150	MAP2	down	1.55	1.51E-03	chr2(+):210288771-210598834	microtubule-associated protein 2	MAP2A, AP2B, AP2	4133	ENSG000000	113.82	73.52
GSHG0027948	ADAP1 // COX19	down	1.54	3.71E-02	chr7(-):937538-1015235	ArfGAP with dual PH domains 1 // cytochrom	GCS1L, GC104475	11033, 0639	ENSG000001	36.81	23.83
GSHG0029420	LONRF1	down	1.54	1.32E-02	chr8(-):12579406-12612992	LON peptidase N-terminal domain and ring fi	FLJ23749, NF191	91694	ENSG000001	37.99	24.65
GSHG0031133	--	down	1.51	2.11E-04	chr9(-):125578333-125579531	--	--	--	--	117.12	77.33
GSHG0012534	TMEM106A	down	1.51	4.74E-02	chr17(+):41363894-41371957	transmembrane protein 106A	MGC20235	113277	ENSG000001	35.63	23.57
GSHG0017713	TGFA	down	1.51	4.31E-05	chr2(-):70674414-70781147	transforming growth factor, alpha	--	7039	ENSG000001	200.10	132.71

Supplementary Table S2 : SUV39H2 target genes differentially regulated by the SUV39H2 isoforms.
List of genes differentially expressed in HeLa cells overexpressing either SUV39H2-L or SUV39H2-S isoforms. Transcriptome wide analysis was performed as described in the materials and methods sections. The transcriptional changes for each gene is calculated as the ratio of the SUV39H2-S versus the SUV39H2-L transcriptional intensities The list contains the genes displaying a fold change higher than 1.5 and a p-value <0.05. H19 was also retained as a gene regulated in a H3K9me3-dependent manner.

Gene Name	Accesion Number	Ensembl transcript ID	Forward primer location	Forward primer sequence	Reverse Primer location	Reverse Primer sequence	Amplicon size	Use	RT priming
human G9a	14129	ENST00000375537	ex9	GCAAGGCCAAGAAGAAATGGCGAA	ex11	TCTCGCTGATGCGGTCAATCTT	303 bp or 201 bp according ex10 inclusion	RT-PCR	oligo dT
			ex5	AGTGATGATGTCCACTCACTGGGA	ex7	AGAGACTGAAGTCATCACCCACCA	187 bp	RT-PCR	oligo dT
mouse G9a	MGI:2148922	ENSMUST00000114033	ex9	GCGCAAGGCCAAGAAGAAATG	ex11	CAGTGAAGACGTGTCATTGGAGAC	226 bp or 124 bp according to ex10 inclusion	RT-PCR	1/3RH+2/3oligo dT
human SUV39H2	17287	ENST00000354919	ex2	GTGTGTGCCTTGCCTAGTTTCACT	ex4	CTGTCCTCGTCTTTCAGCTTCTTCAC	850 bp, 310 bp or 178 bp according to ex3 inclusion	RT-PCR	oligo dT
			ex4	ATGAATTCACAGTGGATGCGGCTC	ex6	TGGCTGGGCTGTGGTCAATAGAAT	234 bp	RT-PCR	oligo dT
mouse SUV39H2	MGI:1890396	ENSMUST00000027956	ex2	GCGCAAGGCCAAGAAGAAATG	ex4	CAGTGAAGACGTGTCATTGGAGAC	880 bp or 220 bp according to ex10 inclusion	RT-PCR	1/3RH+2/3 specific reverse primer in ex5 : GGCAGCCGAGTATCAAGGTTATC
chicken SUV39H2		ENSGALT00000029187	ex2	GTATGTGCCATGTCTGGCTTCAC	ex4	CGTCTCTCTGCTTCCTCACTTGTA	843 bp or 171 bp according to ex3 inclusion	RT-PCR	1/3RH+2/3oligo dT
zebrafish SUV39H1b		ENSDART00000078152	ex2	CACAGATGGGAAGAGGCAAACA	ex4	CGTCCACATAATCCAGGTCAAACA	770 bp or 116 bp according to ex3 inclusion	RT-PCR	1/3RH+2/3oligo dT
human RPLP0	10371	ENST00000228306	ex6	AGGTGTTCGACAATGGCAGCAT	ex7	TGCAGACAGACACTGGCAACAT	112 bp	RT-PCR	oligo dT
			proximal promoter	ACAGAGCGACACTCCGTCTCAAA	proximal promoter	ACCTGGCGAGCTCAGCAAACTAAA	94 bp	ChIP-PCR	
human GAPDH	4141	ENST00000229239	ex4	TCCCATCACCATCTTCCAGG	ex5	CATCGCCCCACTTGATTTTG	53 bp	RT-PCR	oligo dT
human L1112	RP11-1112C15.1	ENSG00000205293	ex2	AGAAATATACAGGCATACTTGTGG	ex2/ex3	TGCACTGTATTTTTCCACATGTTG	83 bp	RT-PCR	oligo dT
human CD2	1639	ENST00000369478	ex3	CAGGGAACAAAGTCAGCAAGGAA	ex4	CAGTGCCACAAAGACCATCAAGAG	125 bp	RT-PCR	oligo dT
			proximal promoter	CTGTGTGGTTGAATTTGGCTTCTTG	proximal promoter	GCCCTTCTGATGTGCTCCTTT	196 bp	ChIP-PCR	
human DHRS2	18349	ENST00000557535	ex2	CCAGTGAGCAGATCTGGGACAA	ex3	TTCTCCATGTAGGGCAGCAACT	88 bp	RT-PCR	oligo dT
			ex1	CGAATGCACCGAGAGGAATGAA	ex1	GATGGTCTCACCACTTGGCAATAG	215 bp	ChIP-PCR	
human ACTL8	24018	ENST00000375406	ex2	ATCATCACGGAGACACCCTTGA	ex3	CATAGCCAGAATCAACCACCACTC	163 bp	RT-PCR	oligo dT
			proximal promoter	GCTGCTTTGCGTCTGGTTTCT	proximal promoter	ATGTGGAGCGGAGAGGCAATAA	143 bp	ChIP-PCR	
human MAGEB6	23796	ENST00000379034	ex1	GTCCCAGATCTTCTCAGCTGTCTT	ex2	CTCACAGGTACGGAGCTTACTCTT	125 bp	RT-PCR	oligo dT
			proximal promoter	CAACTCTGGACTCTCAGGGAATGA	proximal promoter	GGACCCTTGGACTTCTCTATCTGTT	81 bp	ChIP-PCR	
human CCL5	10632	ENST00000293272	ex2	CGTGCCCACATCAAGGAGTATTT	ex3	CAAGGACTCTCCATCCTAGCTCAT	162 bp	RT-PCR	oligo dT
			proximal promoter	CTCAGTGACTTCTGATCCTGTCCTAAC	proximal promoter	TCTCCCTCACTGCTCTCTCATCTA	193 bp	ChIP-PCR	
human CCN1	2155	ENST00000252456	ex1	ACATGTGAGGAGGGAAGAGTGT	ex1	TCGGTTGAAGTGAGCAGAGGA	123 bp	RT-PCR	oligo dT
			proximal promoter	CCTGCTTCAAGACTCCAGAGAGATA	proximal promoter	GTGATGTCAGGCCCTTGGTATTG	207 bp	ChIP-PCR	
human H19	4713	ENST00000414790	ex1	AGTGGACTTGGTGACGCTGTAT	ex1	TGGATGCTGTACTGTCTGCCAA	73 bp	RT-PCR / ChIP	oligo dT
human KRT15	3866	ENSG00000171346	ex2	GAGGGCCTGAATGAGGAGCTA	ex3	TGAAGAACCAGGCCTCGACAT	205 bp	RT-PCR	1/3RH+2/3oligo dT
			ex1	TGCTGACCTGGAGGTGAAGAT	ex1	GAAGTATTGGCTGTAGTCGCATTCTG	82 bp	ChIP-PCR	
human CCL11	6356	ENST00000305869	ex1	AGGCTGAGACCAACCCAGAAA	ex2	TCCTGTAGCTCTCTAGTCGCTGAA	214 bp	RT-PCR	1/3RH+2/3oligo dT
			ex2	GCGACTAGAGAGCTACAGGAGAAT	ex3	ACTCAGGCTCTGGTTTGGTTTC	200 bp	RT-PCR	1/3RH+2/3oligo dT
			proximal promoter	TTGGTTTCCTTGCTCCTTTCCC	proximal promoter	ACAGAAGAGAGGCAAGCTAGTAGG	200 bp	ChIP-PCR	
human C5orf46	389336	ENST00000318315	ex1	GCTACCAGAATGGCTGTCTCAGTA	ex2	GGAAGTCTGGCTTTGGGTCTTTG	145 bp	RT-PCR	1/3RH+2/3oligo dT
			proximal promoter	GAAGAAGGAAGAGCAAGAGCAGAC	proximal promoter	CCAAGCAGCAGGCAACAAATC	151 bp	ChIP-PCR	
human IL10RB	3588	ENSG00000243646	ex2	GCCAAAGGGAACCTGACTTTCACA	ex3	ATTCAGCCCTGACTCTCAAGGTGT	142 bp	RT-PCR	1/3RH+2/3oligo dT
human VTN	7448	ENST00000573054	ex6	GAGGACATCTTCGAGCTTCTCTTCTG	ex7	GGTTGCGATGCCTAAACCTTTGT	130 bp	RT-PCR	1/3RH+2/3oligo dT
human RALY	15921	ENST00000375114	ex1	CGGCTTCCTCCAGACCTC	ex2	TGAGCTTAGCCAGCTGTTTC	98 bp		
human HNRPA1	5031	ENST00000550482	ex5	GCAAAACCACGAAACCAAG	ex6	AAAATTATGTCAACACACAAAAAGG	114 bp	RT-qPCR	1/3RH+2/3oligo dT
human RBM9	9906	ENST00000405409	ex7	TGGAAATTAAGCCCAGTAGTTG	ex8	TGATACCCCCTCTTCCTGATAG	124 bp	RT-qPCR	1/3RH+2/3oligo dT
human RBM39	15923	ENST00000361162	ex7	GACCAAGGGATTTGGAAGAG	ex8	TAAGCAATTCCTTTGGAACG	100 bp	RT-qPCR	1/3RH+2/3oligo dT
human SRp20	10785	ENST00000373715	ex1	AGCGTGTGGATTTGAGCC	ex2	CATTGTTTCCAAGATTGCCT	107 bp	RT-qPCR	1/3RH+2/3oligo dT
human TRA2B	10781	ENST00000453386	ex4	CTAGGCGTTCAAGAGGATTTG	ex5	GTGTTGGCGTATGTGGTCTT	147 bp	RT-qPCR	1/3RH+2/3oligo dT
human SAM68	18116	ENST00000327300	ex1	GTCCTTCACTCACGCCAT	ex2	AAATAAATCCAAGTAATTCTCCTCATC	100 bp	RT-qPCR	1/3RH+2/3oligo dT

Name	name strand	siRNA location	siRNA 5'->3'	overhanged	target DNA (5'->3')
siRNA_Control	sense	non targetting	UAGCAAUGACGAAUGCGUA	dTdT	TAGCAATGACGAATGCGTA
	antisense		UACGCAUUCGUCAUUGCUA	dTdT	
siRNA_G9a_1	sense	ex11-ex12	GGAGAGCUGUCAGGCUGCA	dTdT	GGAGAGCTGTCAGGCTGCA
	antisense		UGCAGCCUGACAGCUCUCC	dTdT	
siRNA_G9a_2	sense	ex24-ex25	GCAUCAAGGUGCGGCUACA	dTdT	GCATCAAGGTGCGGCTACA
	antisense		UGUAGCCGCACCUUGAUGC	dTdT	
siRNA_SUV39H2_1	sense	ex4-ex5	CAGCUGUGACCCAAAUCUU	dTdT	CAGCTGTGACCCAAATCTT
	antisense		AAGAUUUGGGUCACAGCUG	dTdT	
siRNA_SUV39H2_2	sense	ex5-ex6	GAAAGGUUCUGGAGAUAUA	dTdT	GAAAGGTTCTGGAGATATA
	antisense		UAUAUCUCCAGAACCUUUC	dTdT	
siRNA_SUV39H2_e2e4	sense	ex2-ex4	GUAGUAAAGGUAAUCACAA	dTdT	GTAGTAAAGGTAATCACAA
	antisense		UUGUGAUUACCUUUACUAC	dTdT	
siRNA_SUV39H2_e3Se4	sense	ex3S-ex4	CUCAGGUAAUCACAAGUGA	dTdT	CTCAGGTAATCACAAGTGA
	antisense		UCACUUGUGAUUACCUGAG	dTdT	
siRNA_SUV39H2_e3Le4	sense	ex3L-ex4	GUUGGAGAGGUAAUCACAA	dTdT	GTTGGAGAGGTAATCACAA
	antisense		UUGUGAUUACCUCUCCAAC	dTdT	
siRNA_HNRNPA1	sense	ex3	CUUUGGUGGUGGUCGUGGA	dTdT	CTTTGGTGGTGGTCGTGGA
	antisense		UCCACGACCACCACCAAAG	dTdT	
siRNA_SAM68	sense	ex5	UGAUGGAUGAUAUCUGUCA	dTdT	TGATGGATGATATCTGTCA
	antisense		UGACAGAUAUCAUCCAUCA	dTdT	
siRNA_RALY	sense	ex3	CUCUCGAGUCUUCAUUGGA	dTdT	CTCTCGAGTCTTCATTGGA
	antisense		UCCAAUGAAGACUCGAGAG	dTdT	
siRNA_RBM9	sense	ex3	CAGACACAAAGUAGUGAAA	dTdT	CAGACACAAAGTAGTGAAA
	antisense		UUUCACUACUUUGUGUCUG	dTdT	
siRNA_RBM39	sense	ex7	GAUGCAAGGACAGUCUUCU	dTdT	GATGCAAGGACAGTCTTCT
	antisense		AGAAGACUGUCCUUGCAUC	dTdT	
siRNA_SRp20	sense	ex2	GCAUCGUGAUUCCUGUCCA	dTdT	GCATCGTGATTCCTGTCCA
	antisense		UGGACAGGAAUCACGAUGC	dTdT	
siRNA_TRA2B	sense	ex3	GUCUUACAGUCGAGAUUAU	dTdT	GTCTTACAGTCGAGATTAT
	antisense		AUAAUCUCGACUGUAAGAC	dTdT	

Supplementary Table S3 : Sequences of primers for PCR and sequences of siRNAs

Sample	Raw reads	Average base quality over 30	Sequence length	%GC	Total mapped reads
pLVX_1	57,683,411	100%	50	51	(93.5%) 53,336,056
pLVX_2	55,270,068	100%	50	50	(96.1%) 52,723,331
pLVX_3	42,509,141	100%	50	50	(96.2%) 40,592,080
G9a_e10_1	39,316,485	100%	50	51	(93.0%) 36,084,509
G9a_e10_2	56,272,391	100%	50	50	(96.4%) 53,851,594
G9a_e10_3	29,995,057	100%	50	50	(96.6%) 28,768,149
G9a_Δe10_1	30,951,069	100%	50	51	(96.8%) 29,729,406
G9a_Δe10_2	44,665,122	100%	50	50	(96.7%) 42,872,820
G9a_Δe10_3	47,980,571	100%	50	50	(96.9%) 46,160,588
SUV39H2_e3L_1	35,387,857	100%	50	50	(96.2%) 33,809,030
SUV39H2_e3L_2	36,529,662	100%	50	50	(96.0%) 34,815,356
SUV39H2_e3L_3	65,932,495	100%	50	50	(96.0%) 62,871,033
SUV39H2_e3S_1	26,309,193	100%	50	50	(96.2%) 25,122,569
SUV39H2_e3S_2	49,701,123	100%	50	51	(96.2%) 47,465,352
SUV39H2_e3S_3	53,407,232	100%	50	50	(96.2%) 51,009,422

Supplementary Table S4 : RNA-Seq statistics

SUPPLEMENTARY REFERENCES

59. Medjkane,S., Novikov,E., Versteege,I. and Delattre,O. (2004) The tumor suppressor hSNF5/INI1 modulates cell growth and actin cytoskeleton organization. Cancer Res., 64, 3406–3413.
60. Mertineit,C., Yoder,J.A., Taketo,T., Laird,D.W., Trasler,J.M. and Bestor,T.H. (1998) Sex-specific exons control DNA methyltransferase in mammalian germ cells. Development, 125, 889–897.
61. Bonfils,C., Beaulieu,N., Chan,E., Cotton-Montpetit,J. and MacLeod,A.R. (2000) Characterization of the Human DNA Methyltransferase Splice Variant Dnmt1b. J. Biol. Chem., 275, 10754–10760.
62. Robertson,K.D., Uzvolgyi,E., Liang,G., Talmadge,C., Sumegi,J., Gonzales,F.A. and Jones,P.A. (1999) The human DNA methyltransferases (DNMTs) 1, 3a and 3b: coordinate mRNA expression in normal tissues and overexpression in tumors. Nucleic Acids Res., 27, 2291–2298.
63. Gopalakrishnan,S., Emburgh,B.O.V., Shan,J., Su,Z., Fields,C.R., Vieweg,J., Hamazaki,T., Schwartz,P.H., Terada,N. and Robertson,K.D. (2009) A Novel DNMT3B Splice Variant Expressed in Tumor and Pluripotent Cells Modulates Genomic DNA Methylation Patterns and Displays Altered DNA Binding. Mol. Cancer Res., 7, 1622–1634.
64. Van Emburgh,B.O. and Robertson,K.D. (2011) Modulation of Dnmt3b function in vitro by interactions with Dnmt3L, Dnmt3a and Dnmt3b splice variants. Nucleic Acids Res., 39, 4984–5002.
65. Matsuda,H., Paul,B.D., Choi,C.Y. and Shi,Y.-B. (2007) Contrasting Effects of Two Alternative Splicing Forms of Coactivator-Associated Arginine Methyltransferase 1 on Thyroid Hormone Receptor-Mediated Transcription in Xenopus laevis. Mol. Endocrinol., 21, 1082–1094.
66. Ohkura,N., Takahashi,M., Yaguchi,H., Nagamura,Y. and Tsukada,T. (2005) Coactivator-associated Arginine Methyltransferase 1, CARM1, Affects Pre-mRNA Splicing in an Isoform-specific Manner. J. Biol. Chem., 280, 28927–28935.
67. Wang,L., Charoensuksai,P., Watson,N.J., Wang,X., Zhao,Z., Coriano,C.G., Kerr,L.R. and Xu,W. (2013) CARM1 automethylation is controlled at the level of alternative splicing. Nucleic Acids Res., 41, 6870–6880.
68. Pruitt,K., Zinn,R.L., Ohm,J.E., McGarvey,K.M., Kang,S.-H.L., Watkins,D.N., Herman,J.G. and Baylin,S.B. (2006) Inhibition of SIRT1 reactivates silenced cancer genes without loss of promoter DNA hypermethylation. PLoS Genet., 2, e40.
69. Lynch,C.J., Shah,Z.H., Allison,S.J., Ahmed,S.U., Ford,J., Warnock,L.J., Li,H., Serrano,M. and Milner,J. (2010) SIRT1 Undergoes Alternative Splicing in a Novel Auto-Regulatory Loop with p53. PLoS ONE, 5, e13502.
70. Shah,Z.H., Ahmed,S.U., Ford,J.R., Allison,S.J., Knight,J.R.P. and Milner,J. (2012) A Deacetylase-Deficient SIRT1 Variant Opposes Full-Length SIRT1 in Regulating Tumor Suppressor p53 and Governs Expression of Cancer-Related Genes. Mol. Cell. Biol., 32, 704–716.
71. Yang,W., Xia,Y., Hawke,D., Li,X., Liang,J., Xing,D., Aldape,K., Hunter,T., Alfred Yung,W.K. and Lu,Z. (2012) PKM2 Phosphorylates Histone H3 and Promotes Gene Transcription and Tumorigenesis. Cell, 150, 685–696.
72. Zibetti,C., Adamo,A., Binda,C., Forneris,F., Toffolo,E., Verpelli,C., Ginelli,E., Mattevi,A., Sala,C. and Battaglioli,E. (2010) Alternative Splicing of the Histone Demethylase LSD1/KDM1 Contributes to the Modulation of Neurite Morphogenesis in the Mammalian Nervous System. J. Neurosci., 30, 2521–2532.
73. Toffolo,E., Rusconi,F., Paganini,L., Tortorici,M., Pilotto,S., Heise,C., Verpelli,C., Tedeschi,G., Maffioli,E., Sala,C., et al. (2013) Phosphorylation of neuronal Lysine-Specific

Demethylase 1LSD1/KDM1A impairs transcriptional repression by regulating interaction with CoREST and histone deacetylases HDAC1/2. J. Neurochem., 10.1111/jnc.12457.
74. Kwon,S.Y., Xiao,H., Wu,C. and Badenhorst,P. (2009) Alternative splicing of NURF301 generates distinct NURF chromatin remodeling complexes with altered modified histone binding specificities. PLoS Genet., 5, e1000574.
75. Grzenda,A., Lomberk,G., Svingen,P., Mathison,A., Calvo,E., Iovanna,J., Xiong,Y., Faubion,W. and Urrutia,R. (2013) Functional characterization of EZH2beta reveals the increased complexity of EZH2 isoforms involved in the regulation of mammalian gene expression. Epigenetics Chromatin, 6, 3.
76. Yeo,G. and Burge,C.B. (2004) Maximum entropy modeling of short sequence motifs with applications to RNA splicing signals. J. Comput. Biol. J. Comput. Mol. Cell Biol., 11, 377–394.
